# 21-Hydroxylase-Specific CD8+ T Cells in Autoimmune Addison’s Disease Are Restricted by HLA-A2 and HLA-C7 Molecules

**DOI:** 10.3389/fimmu.2021.742848

**Published:** 2021-10-14

**Authors:** Alexander Hellesen, Sigrid Aslaksen, Lars Breivik, Ellen Christine Røyrvik, Øyvind Bruserud, Kine Edvardsen, Karl Albert Brokstad, Anette Susanne Bøe Wolff, Eystein Sverre Husebye, Eirik Bratland

**Affiliations:** ^1^ Department of Clinical Science, University of Bergen, Bergen, Norway; ^2^ KG Jebsen Centre for Autoimmune Diseases, University of Bergen, Bergen, Norway; ^3^ Department of Medicine, Haukeland University Hospital, Bergen, Norway; ^4^ Broegelmann Research Laboratory, University of Bergen, Bergen, Norway; ^5^ Department of Medical Genetics, Haukeland University Hospital, Bergen, Norway

**Keywords:** CD8+ T cells, 21-hydroxylase, autoimmune, Addison’s disease, epitopes

## Abstract

**Objectives:**

CD8+ T cells targeting 21-hydroxylase (21OH) are presumed to play a central role in the destruction of adrenocortical cells in autoimmune Addison’s disease (AAD). Earlier reports have suggested two immunodominant CD8+ T cell epitopes within 21OH: LLNATIAEV (21OH_342-350_), restricted by HLA-A2, and EPLARLEL (21OH_431-438_), restricted by HLA-B8. We aimed to characterize polyclonal CD8+ T cell responses to the proposed epitopes in a larger patient cohort with AAD.

**Methods:**

Recombinant fluorescent HLA-peptide multimer reagents were used to quantify antigen-specific CD8+ T cells by flow cytometry. Interferon-gamma (IFNγ) Elispot and biochemical assays were used to functionally investigate the 21OH-specific T cells, and to map the exactly defined epitopes of 21OH.

**Results:**

We found a significantly higher frequency of HLA-A2 restricted LLNATIAEV-specific cells in patients with AAD than in controls. These cells could also be expanded *in vitro* in an antigen specific manner and displayed a robust antigen-specific IFNγ production. In contrast, only negligible frequencies of EPLARLEL-specific T cells were detected in both patients and controls with limited IFNγ response. However, significant IFNγ production was observed in response to a longer peptide encompassing EPLARLEL, 21OH_430-447_, suggesting alternative dominant epitopes. Accordingly, we discovered that the slightly offset ARLELFVVL (21OH_434-442_) peptide is a novel dominant epitope restricted by HLA-C7 and not by HLA-B8 as initially postulated.

**Conclusion:**

We have identified two dominant 21OH epitopes targeted by CD8+ T cells in AAD, restricted by HLA-A2 and HLA-C7, respectively. To our knowledge, this is the first HLA-C7 restricted epitope described for an autoimmune disease.

## Introduction

Patients with autoimmune Addison’s disease (AAD) suffer from adrenocortical insufficiency; an inability to produce adequate amounts of steroid hormones due to immune-mediated destruction of hormone-producing cells in the adrenal cortex. This potentially lethal disease can occur in isolation or as a component of autoimmune polyendocrine syndrome types 1 (APS-1) or 2 (APS-2), the former being a monogenic disorder caused by mutations in the *AIRE* gene (1). Circulating autoantibodies against 21-hydroxylase (21OH), a steroidogenic enzyme of the adrenal cortex, serve as a biomarker of the disease (2, 3). Autoantibodies against 21OH are often detectable prior to the development of clinical disease and correlate with the degree of adrenal dysfunction (4, 5), but their role in the disease pathogenesis remains obscure (6). Presumably, the progressive destruction of adrenocortical cells in AAD is mediated by infiltrating autoreactive T cells. Accordingly, strong genetic associations are found between AAD and the HLA locus, in particular the HLA class II allelic combination DR3/DQ2-DR4/DQ8 (2, 7). Among HLA class I genes, the B*0801 allele occurs more frequently in patients than controls (8) and increases the susceptibility to AAD in combination with DR3 (9). Furthermore, a recently performed genome-wide association study (GWAS) has revelated AAD risk loci in several T cell related genes, including *AIRE*, *CTLA4* and *PTPN22*, supporting the notion of T cell-driven pathology (10).

T cell responses to 21OH in patients with AAD were first demonstrated by Bratland et al. in the form of proliferation and IFNγ secretion by peripheral blood mononuclear cells (PBMCs) in response to 21OH whole protein and selected peptides (11). Since then, epitope mapping has suggested the presence of two immunodominant peptides recognized by CD8^+^ T cells. IFNγ production by PBMCs in response to a C-terminal peptide of 21OH was described in AAD patients but not in healthy controls (12). These responses were proposed to target the HLA-B8-restricted octameric sequence EPLARLEL (position 21OH_431-438_), as most of the responding patients carried the HLA-B*0801 allele. More recently, Dawoodji et al. reported on a HLA-A2-restricted epitope (LLNATIAEV, position 21OH_342-350_), and demonstrated the ability of a 21OH_342-350_-specific CD8^+^ T cell clone to lyse LLNATIAEV-pulsed target cells by granzyme B release (13). In the two studies, the physical detection of EPLARLEL- and LLNATIAEV-specific CD8^+^ T cells were demonstrated by HLA-tetramer staining in only two and one patient(s), respectively. Furthermore, T cell responses to the exact octameric or nonameric epitope were only shown in the latest study using a LLNATIAEV-specific T cell line or clone prepared from one individual patient. In contrast, the reported IFNγ responses for the respective patient cohorts were based on 21OH peptides of 18-20 amino acids length (21OH_337-354_ and 21OH_431-450_, respectively).

In the present study, we aimed to characterize polyclonal CD8^+^ T cell responses to the proposed defined epitopes LLNATIAEV (21OH_342-350_) and EPLARLEL (21OH_431-438_) in a larger patient cohort with AAD. In contrast to LLNATIAEV, which was clearly targeted by T cells from HLA-A2 positive patients, no CD8^+^ T cell responses could be detected against EPLARLEL. Further scrutiny led us to discover a new immunodominant epitope, ARLELFVVL (21OH_434-442_), presented by HLA-C*0701, the first reported in an autoimmune disease.

## Materials and Methods

### Patients and Controls

Heparinized peripheral blood was obtained from 34 patients with verified primary adrenocortical insufficiency due to AAD, included in the Norwegian National Registry for Organ-Specific Autoimmune Diseases (ROAS). Patient characteristics are summarized in **Supplementary Table 1**. Serving as controls, peripheral blood was obtained from 27 healthy blood donors (C1-C6, C9, and C11-C30) from the Blood Bank at Haukeland University Hospital (Bergen, Norway), and three individuals with other (non-adrenal) autoimmune diseases (C7, C8 and C10) included in ROAS. The study was approved by the Regional Committee for Medical and Health Research Ethics, project number 2018/1417. All patients and controls from ROAS had given informed consent approved by the Health Region West ethics committee (147/96-47·96). Healthy individuals had given informed consent at the Blood Bank.

Autoantibodies against 21OH were measured by routine radioimmunoassays as described by Oftedal et al. (14), except that dithiothreitol was omitted from the sample buffer. Positivity was defined as a threshold index ration based on a pool of 200 blood donor sera and a positive control serum from an AAD patient containing high levels of 21OH autoantibodies. All AAD patients were positive for autoantibodies against 21OH, confirming autoimmune etiology.

The expression of HLA-A2 and HLA-B8 molecules was determined by flow cytometry on lysed whole blood or PBMCs using FITC conjugated anti-human HLA-A2 (Biolegend, clone BB7.2) and APC conjugated anti-human HLA-B8 (Miltenyi Biotec, clone REA145) antibodies, respectively. Genotyping of HLA-C7 alleles was performed by PCR using sequence-specific primers as described by Bunce et al. (15). For the majority of the patients and a subgroup of the controls, the presence of specific HLA alleles could be confirmed by other genotyping methods from other studies, such as sequence-specific oligonucleotide probe system (8) and allelic imputation from genome-wide array genotyping (10).

### Peptides and MHC Multimers

Custom designed, PE-labeled HLA-A*0201 and HLA-B*0801 dextramers containing the LLNATIAEV (A2*LLNATIAEV) and EPLARLEL (B8*EPLARLEL) peptides, respectively, were purchased from Immudex. Custom designed PE-labeled HLA-C*0701 streptamer containing the ARLELFVVL (C7*ARLELFVVL) peptide was purchased from IBA Lifesciences. Purified LLNATIAEV and EPLARLEL peptides were purchased from Proimmune/thinkpeptides. Peptides used for epitope mapping were purchased from GenScript and included the long C-terminal peptide G**EPLARLEL**FVVLTRLLQ (Pep34, 21OH_430-447_) and ten consecutively overlapping 9-mer peptides covering the same sequence (Pep1-Pep10). All peptides were reconstituted in cell culture-grade DMSO at 10-40 mg/mL to obtain stock solutions and diluted in RPMI-1640 (Life Technologies) prior to use.

### PBMC Stimulation and Expansion

**Figure 1:**
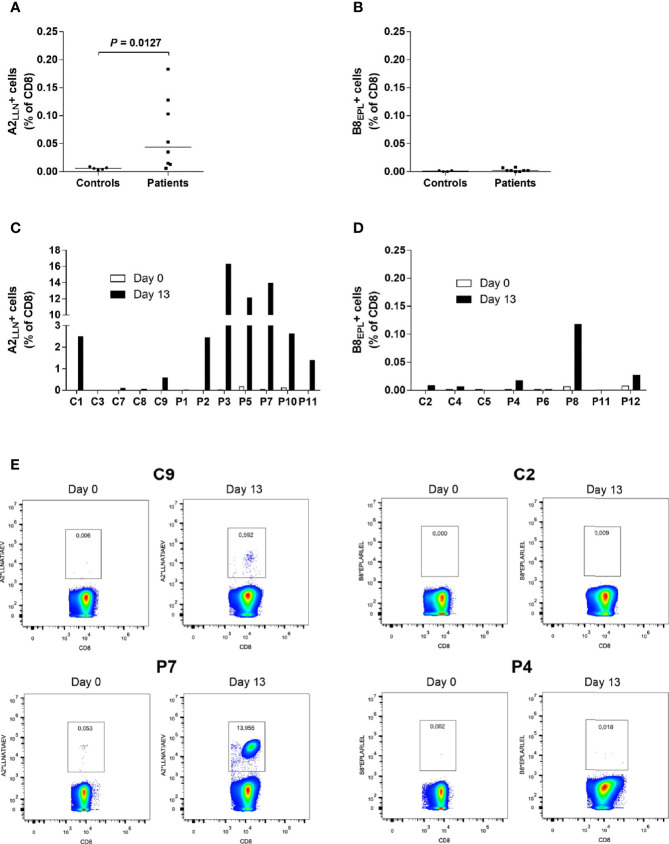
Increased frequencies of LLNATIAEV-specific CD8+T cells in AAD patients. **(A)** PBMCs from HLA-A2 positive AAD patients (n = 8) and controls (n = 5) were stained with A2*LLNATIAEV dextramers *ex vivo* and analyzed with flow cytometry. **(B)** PBMCs from HLA-B8 positive AAD patients (n = 8) and controls (n = 4) were stained with B8*EPLARLEL dextramers *ex vivo* and analyzed with flow cytometry. **(C)** PBMCs from HLA-A2 AAD patients (n = 7) and controls (n = 5) were stained with A2*LLNATIAEV dextramers following 13 days of peptide-induced *in vitro* expansion and analyzed with flow cytometry. Differences were not statistically significant. **(D)** PBMCs from HLA-B8 AAD patients (n = 5) and controls (n = 3) were stained with B8*EPLARLEL dextramers following 13 days of peptide-induced *in vitro* expansion and analyzed with flow cytometry. The graphs **(A–D)** show the frequencies of dextramer-binding CD8+ T cells. Lines **(A, B)** represent median values. Statistical analyses were performed using Mann-Whitney U-test; P-values <0.05 are shown. **(E)** Representative flow cytometry dextramer plots from two HLA-A2 (C9, P7) and two HLA-B8 (C2, P4) individuals *ex vivo* and following expansion (day 13). A strict, fixed gate was used to distinguish dextramer-positive from negative cells. Gate numbers represent the frequencies (percentage) of dextramer-positive cells among total CD8+ T cells. Statistical analyses were performed using Mann-Whitney U-test; P-values <0.05 are shown.

**Figure 2:**
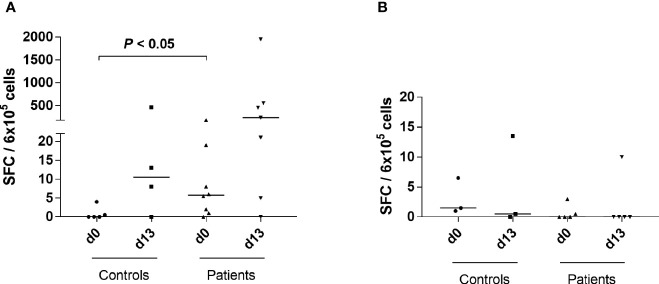
Increased IFNγ responses to LLNATIAEV, but not EPLARLEL, in patients with AAD. **(A)** PBMCs from HLA-A2 positive individuals were stimulated with LLNATIAEV peptide *ex vivo* (AAD patients n = 8, controls n = 5) or restimulated following 13 days of peptide-induced *in vitro* expansion (AAD patients n = 7, controls n = 4). **(B)** PBMCs from HLA-B8 positive individuals were stimulated with EPLARLEL peptide *ex vivo* (AAD patients n = 5, controls n = 3) or restimulated following 13 days of peptide-induced *in vitro* expansion (AAD patients n = 5, controls n = 3). The number of IFNγ-secreting cells (SFC) was measured by ELISPOT and is expressed as SFC per 6x10^5^ PBMCs. Lines represent median values. Statistical analyses were performed using Mann-Whitney U-test; P-values <0.05 are shown.

**Figure 3:**
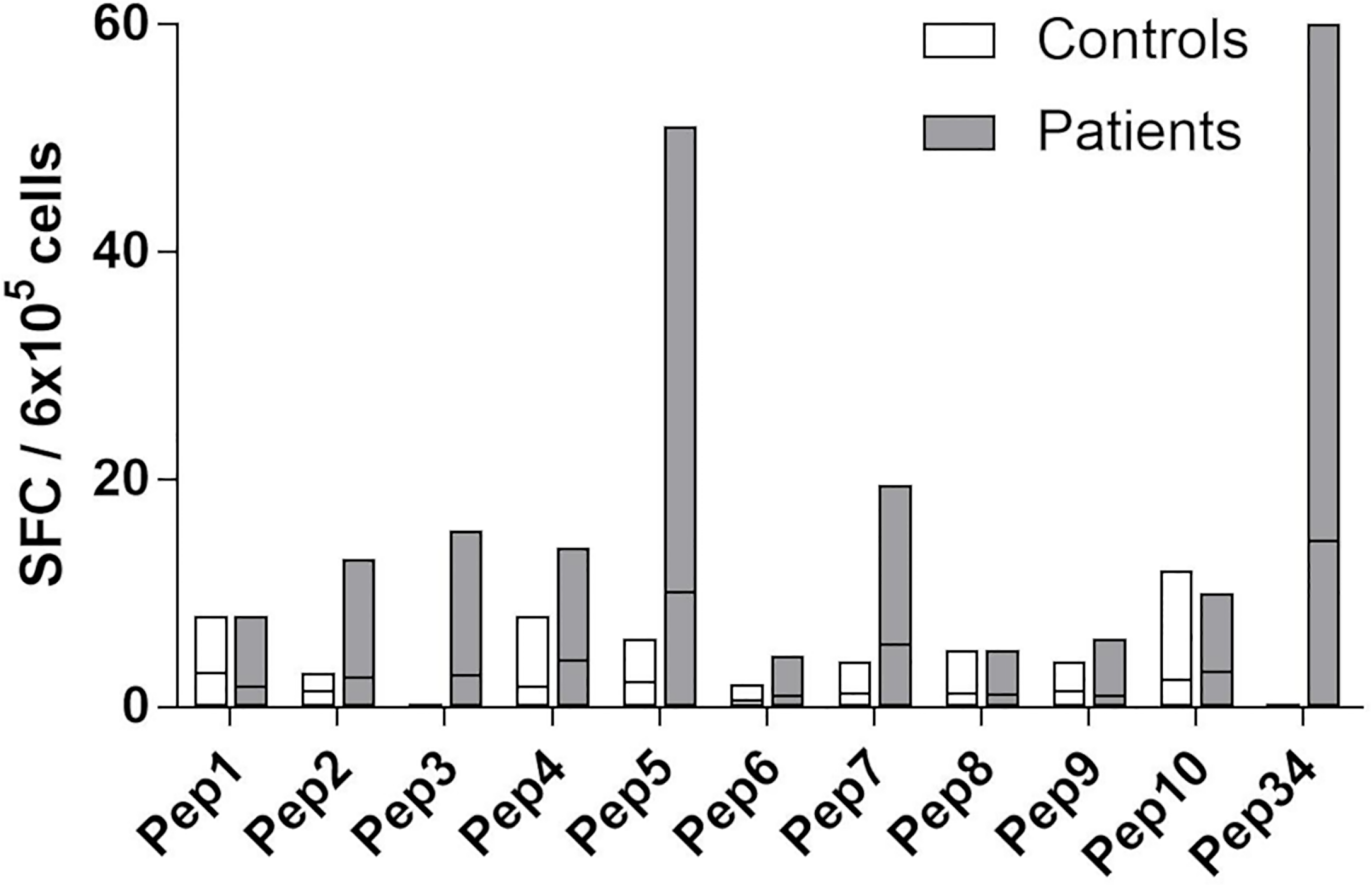
Fine-mapping of T cell responses towards Pep34 (21OH_430-447_ GEPLARLELFVVLTRLLQ). PBMCs from patients (n = 10) and controls (n =5) were stimulated *ex vivo* with individual overlapping 9-mer (Pep1-10) peptides covering Pep34, as well as Pep34 itself, and subjected to IFNγ ELISPOT. Of note, Pep2 represents EPLARLELF. Results are expressed as IFNγ SFCs per 6x10^5^ PBMCs and lines represent median values.

**Figure 4:**
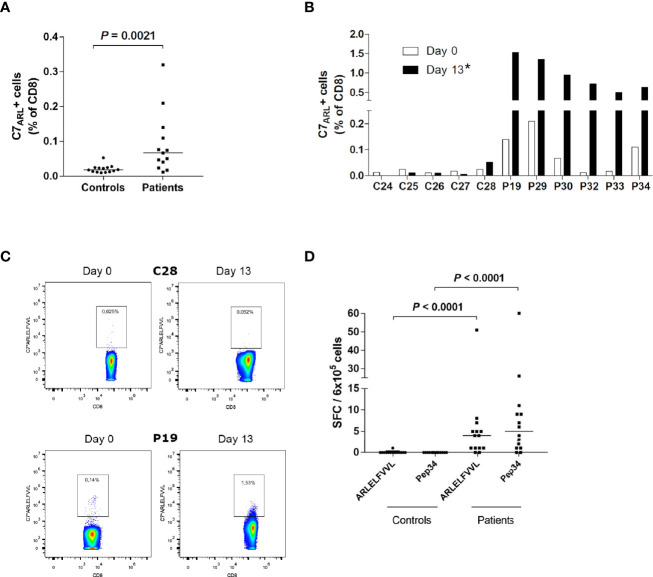
IFNγ producing ARLELFVVL-specific CD8+ T cells are enriched in AAD patients. **(A)** PBMCs from HLA-C7-positive AAD patients (n = 13) and controls (n = 14) were stained with C7*ARLELFVVL streptamers *ex vivo* and analyzed with flow cytometry. **(B)** PBMC from HLA-C7-positive AAD patients (n = 6) and controls (n = 5) were stimulated with ARLELFVVL peptide and expanded for 13 days *in vitro*, before being stained with C7*ARLELFVVL streptamers and analyzed with flow cytometry. The graphs **(A, B)** show the frequencies of streptamer-binding CD8+ T cells. **(C)** Examples of flow cytometry streptamer plots from HLA-C7 individuals (C28, P19) *ex vivo* and following expansion (day 13). A strict, fixed gate was used to distinguish streptamer-positive from negative cells. Gate numbers represent the frequencies (percentage) of streptamer-positive cells among total CD8+ T cells. **(D)** PBMCs from AAD patients (n =14) and controls (n = 14) were stimulated with ARLELFVVL or Pep34 *ex vivo* to detect IFNγ-SFCs. The amount was measured by ELISPOT and is expressed as SFCs per 6x10^5^ PBMCs after background subtraction. Lines represent median values **(A, D)**. Statistical analyses were performed using Mann-Whitney U-test; P-values <0.05 are shown, * in **(B)** signifies a statistically significant difference of streptamer-positive cells between patients and controls on day 13 (*P* = 0.0043).

PBMCs were isolated from heparinized blood by Ficoll-Paque PLUS (GE Healthcare) density gradient centrifugation. Fresh samples were available from five of the patients, while cryopreserved cells (preserved in 90% AB serum/10% DMSO (Sigma-Aldrich) at -150°C) were used for the remaining patients and healthy controls. For some patients and controls, not enough cells were available for all experiments described below. Accompanying tables to **Figures 1**–**4** are available in the Supplementary materials, containing details about which patient and control samples were included for each experiment. PBMCs were cultured in RH-10 medium consisting of RPMI-1640, 10% human AB serum (Sigma-Aldrich), 1% non-essential amino acids, 1% Na-Pyruvate, 1% HEPES, 1% L-glutamine, 1% penicillin-streptomycin (all from Lonza) and 55 µM β-mercaptoethanol (Sigma). On day 0, PBMCs were resuspended in RH-10 medium containing IL-7 (Biolegend, 25 ng/mL) to provide survival signals for naïve and memory T cells (16, 17) and stimulated with 21OH peptides LLNATIAEV, EPLARLEL or ARLELFVVL (10 µg/mL) in 24-well plates at 1.5-3.5x10^6^ cells/mL. On day 3, cells were replenished with fresh RH-10 medium containing IL-2 (Thermo Fisher Scientific, 50 U/mL) and this procedure was repeated every three days thereon until day 9. The cell cultures were split when reaching high confluency (>90%) or when acidification of the medium was evident. For patients P3 and P11 only few LLNATIAEV specific T cells could be demonstrated *ex vivo*. Therefore, an alternative protocol was used where IFNγ-producing cells in response to 6 hour stimulation with LLNATIAEV (day 0) were isolated using an IFNγ Secretion Assay (Miltenyi Biotec), and then expanded with IL-2 as outlined above. On day 13, cells were harvested for dextramer or streptamer staining, and IFNγ ELISPOT was performed for selected samples.

### Dextramer and Streptamer Staining

PBMCs were stained with PE-labeled HLA-A2*LLNATIAEV and/or HLA-B8*EPLARLEL dextramers or HLA-C7*ARLELFVVL streptamers on day 0 and following expansion (day 13). One million PBMCs were stained on day 0 and 4x10^5^-1x10^6^ PBMCs on day 13. The amount of dextramer used for staining was adjusted accordingly. Cells were initially centrifuged at 400 x g before being resuspended in 50 µL 5% FBS/PBS (v/v) and stained with 4-10 µL of dextramers for 10 min at room temperature, or with 4 µl of streptamers for 45 min on ice. The cells were then washed, centrifuged and resuspended in 100-200 µL 5% FBS/PBS, and stained with antibodies towards CD4, CD19, CD14 (all FITC), and CD8 (APC) for 20 min on ice. As part of a test setup, cells from P3 were only stained with dextramers and CD8 antibodies (no negative gate with CD4/CD14/CD19). Finally, cells were washed, centrifuged and resuspended in 200 µL 5% FBS/PBS before being analyzed on an Accuri C6 flow cytometer. The gating strategy is shown in **Supplementary Figure S1**. To reduce false positives, a strict gate was drawn for dextramer/streptamer-positive cells, based upon fluorescence minus one (FMO) controls for each individual sample.

### IFNγ ELISPOT and Cytokine Screening

Quantification of IFNγ-producing cells upon stimulation or restimulation with 21OH peptides was performed with human IFNγ ELISpot^PRO^ or IFNγ ELISpot^PLUS^ kits (Mabtech). On day 0 (*ex vivo*), 6x10^5^ PBMCs were stimulated with LLNATIAEV, EPLARLEL, ARLELFVVL, or Pep34 (10 µg/mL), or with DMSO (0.1% or 0.025%) (blank), in 600 µL RH-10 medium and seeded in triplicates in pre-blocked (RH-10 medium) ELISPOT plates. Plates were incubated for 40h at 37°C before being developed in accordance with the manufacturer’s instructions. Spot-forming cells (SFC) in each experiment were counted manually and independently by two or three individuals.

For LLNATIEV and EPLARLEL, cells from some patients and controls were also tested for IFNγ production on day 13 after expansion. Autologous or syngeneic (HLA-A2 or HLA-B8-matched) PBMCs, depending on the availability of autologous cells, were thawed and subjected to magnetic-activated cell sorting with CD14 and blood dendritic cell antigen-4 (BDCA-4) microbeads (Miltenyi Biotech). The purified population contains monocytes and macrophages (CD14), as well as plasmacytoid dendritic cells (BDCA-4) which possess cross-presenting abilities (18). Sixty thousand CD14^+^/BDCA-4^+^ cells were used as APCs and pulsed with LLNATIAEV or EPLARLEL peptide (2 µg/mL), or with DMSO (blank), for 2 hours at 37°C. Next, 6x10^5^ expanded cells were co-cultured with pulsed APCs in 200 µL RH-10 in pre-blocked ELISPOT plates. For P9, HLA-A2^+^ Priess lymphoblastoid B cells were used as APCs. For P3, 1.5x10^4^ expanded cells were co-cultured with 7.5x10^3^ peptide or DMSO-pulsed autologous APCs. Plates were incubated for 24 hours at 37°C prior to development and counting as described above.

For mapping of alternative epitopes within 21OH_430-447_ sequence, *ex vivo* PBMCs were stimulated with individual overlapping 9-mer peptides and seeded in ELISPOT wells as described above for day 0. The plates were developed and analyzed accordingly.

Supernatants from selected ELISPOT assays at both day 0 and day 13 were harvested and kept at -80°C for Milliplex analyses and/or ELISAs to screen for other relevant cytokines in response to peptide stimulation. A human CD8^+^ T cell premixed multiplex assay comprising the following 17 cytokines was purchased from Millipore (catalogue no: HCDM8MAG15k17PMX): GM-CSF, sCD137, sFas, sFasL, Granzyme A, Granzyme B, IFNγ, IL-2, IL-4, IL-5, IL-6, IL-10, IL-13, MIP-1α, MIP-1β, Perforin and TNFα. Twenty-five microliter samples from ELISPOT assays were run in duplicates or single wells. The assay was performed as recommended by the manufacturer and Luminex 100 (Luminex Corp) and STarStation Software v.3.0 (Applied Cytometry System) was used for recording and analyzing the data. For IL-6 and TNFα, supernatants were also tested with traditional sandwich ELISA (Thermo Fisher Scientific).

### ProImmune REVEAL & ProVE Rapid Epitope Discovery System

To evaluate other potential HLA-B*0801-restricted epitopes within Pep34, we used a custom epitope discovery service offered by ProImmune (19). The service consisted of the following four modules: custom peptide synthesis, MHC-peptide binding assay, MHC pentamer synthesis, and MHC-peptide complex off-rate (t_1/2_) assay. Two sets of overlapping 8-mer and 9-mer peptides covering the Pep34 sequence (GEPLARLELFVVLTRLLQ) were synthesized (module 1) and tested for their ability to bind and stabilize HLA-B*0801 compared to a known T cell epitope (module 2). Candidate binders identified in module 2 were selected for HLA-B*0801 pentamer synthesis (module 3) and the stability (“off-rates”) of the resulting complexes were tested at 6 time points over a 24h period to approximate their half-life (module 4).

### *In Silico* Predictions of HLA-C*0701-Peptide Interactions

The NetMHC 4.0 Server was used to predict binding of 21OH peptides to specific HLA class I molecules (http://www.cbs.dtu.dk/services/NetMHC/) (20, 21). The coordinates for the X-ray solved crystal structure of HLA-C*0702 in complex with the peptide ligand RYRPGTVAL (pdb id: 5VGE) (22), were downloaded from the Protein Data Bank at https://www.rcsb.org. Potential interactions between ARLELFVVL and HLA-C*0701 were modeled by PyMOL Molecular Graphics System v.2.2, with the use of the mutagenesis tool in the software to replace the RYRPGTVAL peptide with ARLELFVVL and to replace Lys90 and Ser123 of the HLA-C molecule with Asn90 and Tyr123, respectively. The APBS electrostatics plugin of PyMOL was used to visualize the electrostatic potential of the peptide binding cleft of HLA-C*0702.

### Protein BLAST Search for Microbial Sequence Homology to CD8+ T Cell Targeted 21OH-Derived Peptides

To determine if proteins from human pathogens contained homologous sequences to 21OH-derived T cell epitopes, we performed a protein BLAST search (https://blast.ncbi.nlm.nih.gov/) using the 9-mer sequences LLNATIAEV and ARLELFVVL. The peptide sequences were blasted against a list of the most common microbial pathogens known to colonize humans in Norway, available from the web pages of the Norwegian Institute of Public Health (https://www.fhi.no/sv/smittsomme-sykdommer/sykdommer/). Only microbes containing homologous sequences to both LLNATIEV and ARLELFVVL were considered. Default settings in the protein BLAST search engine were used. Microbial peptide hits containing gaps were not tolerated.

### Statistics

Comparisons of peptide specific SFC frequencies and dextramer/streptamer frequencies between patients and controls were tested by non-parametric Mann-Whitney U-test using GraphPad Prism v5.02. P-values below 0.05 were considered significant. Only significant P-values are shown in figures.

## Results

### High Frequencies of LLNATIAEV-Specific CD8+ T Cells in AAD Patients

Initial staining (day 0) of PBMCs from AAD patients with highly sensitive MHC dextramers revealed frequencies of LLNATIAEV- and EPLARLEL-specific T cells ranging from 0.002% to 0.183% of total CD8+ T cells (**Figure 1A**). The frequency of LLNATIAEV-specific cells was significantly higher in patients than controls (P = 0.0127). In the patients, LLNATIAEV-specific cells were also more frequent than EPLARLEL-specific cells (P = 0.0018, data not shown). There was no difference in the frequency of EPLARLEL-specific cells between patients and controls (**Figure 1B**). Accordingly, the highest individual frequency observed was for LLNATIAEV-specific cells (P5, 0.183% of CD8+ T cells). Small populations of bright T cells stained with A2*LLNATIAEV were present in five of the patients (P2, P5, P7, P9 and P10), but none of the controls, on day 0. For all individuals expressing both HLA-A2 and HLA-B8, LLNATIAEV-specific cells were more frequent than EPLARLEL-specific cells (**Supplementary Figure 2**), hence LLNATIAEV was chosen as the stimulating peptide in expansion cultures for these patients (except for P11 where both were used).

Following enumeration of peptide-specific CD8+ T cells on day 0, PBMCs were stimulated with LLNATIAEV and/or EPLARLEL peptide in the presence of IL-7. The cells were allowed to expand for 13 days supported by IL-2 supplementation (supplied on day 3, 6 and 9), and then stained with dextramers again (**Figures 1C**–**E**). For P9, there was too few cells for analyses on day 13. Distinct bright populations of peptide-specific cells were evident in 7 out of 11 patients on day 13, including four of the patients that harbored such populations at day 0 (P2, P5, P7 and P10) (**Figure 1C**). Only one patient, P8, presented with a distinct cellular population stained by the B8*EPLARLEL dextramers, but only at frequencies 10-100 fold lower than for A2*LLNATIAEV (**Figure 1D**). The strongest expansion was seen for patients P2, P3, P5, P7 and P10, resulting in frequencies up to 16% of total CD8+ T cells. In the remaining four patients without distinct populations, there were negligible or no increase in the number of dextramer-positive cells. Surprisingly, strong expansion was also evident for C1 and to a certain degree C9, giving rise to populations of LLNATIAEV-specific cells that accounted for 2.5% and 0.5% of CD8+ T cells, respectively. Notably, none of the controls tested positive for 21OH autoantibodies.

### IFNγ Responses to LLNATIAEV (21OH_342-350_)

We followed up previous reports on IFNγ responses to peptides 21OH_337-354_ and 21OH_431-450_ using the shorter, predicted dominant epitopes LLNATIAEV (13) and EPLARLEL (12), respectively. IFNγ ELISPOT assays were performed both *ex vivo* and with expanded cells (**Figures 2A, B**). *Ex vivo* IFNγ SFC counts in response to LLNATIAEV were highest in the patients harboring small populations of A2*LLNATIAEV-staining cells on day 0 (P2, P5, P7, P9 and P10). Although *ex vivo* responses in general were low, a significant difference between patients and controls were noted for IFNγ SFCs (P <0.05, **Figure 2A**). For HLA-B8 carriers, SFC counts rarely exceeded background in response to EPLARLEL stimulation (**Figure 2B**).

After two weeks of expansion with the selected peptides, IFNγ responses seemed confined to patients in which there was clear expansion of LLNATIAEV-specific cells (P2, P3, P5, P7 and P10) (**Figure 2A**). Similarly, LLNATIAEV-specific IFNγ-producing cells were also frequent in control C1 who displayed a distinct population of cells with such specificity on day 13. Again, we could not detect specific responses to EPLARLEL (**Figure 2B**). Thus, IFNγ responses were largely restricted to LLNATIAEV and mainly present in the patients (both *ex vivo* and upon *in vitro* expansion).

Beyond IFNγ, there are few data on the cytokines produced by autoreactive T cells in AAD. In order get insight into such cytokine profiles, we performed a preliminary cytokine screen on supernatants from day 0 and day 13 ELISPOT assays, using a panel of 17 cytokines associated with CD8^+^ T cell responses. Unfortunately, due to limited amounts of material, we were only able to include some of the patients exhibiting peptide-induced expansion and IFNγ responses above background, as well as three control subjects. A complete overview of the results from the entire panel is given in **Supplementary Tables 2**–**7**. Four cytokines appeared to be secreted by cells from several patients, specifically in response to *ex vivo* peptide stimulation on day 0: IL-6, TNFα, MIP-1α and MIP-1β. Some of these data were confirmed by performing individual ELISAs for two of the cytokines; IL-6 and TNFα. **Supplementary Figure 3** displays the results from ELISA (S3A-D) and multiplex (S3E-H) measurements. However, upon restimulation on day 13, virtually all responses faded or were even repressed compared to unstimulated cells. A similar effect was seen on some of the other cytokines in individual patients. However, due to the relative small number of patients and controls and the lack of appropriate replicates for some of the individuals tested, these results must be interpreted with caution.

Still, the overall impression from measuring cytokine responses to 21OH peptides was therefore that IFNγ production as measured by ELISPOT stood out as the most reliable cytokine biomarker for peptide-specific CD8^+^ T cells, in particular for expanded cells.

### Investigation of Alternative Epitopes

Our dextramer and cytokine data (**Figures 1**, **2** and **Supplementary Figure S2**) did not support previous reports suggesting EPLARLEL to be a major CD8^+^ T cell epitope in AAD. Most of the data from these publications were from stimulation experiments with longer peptides (21OH_431-450_ and 21OH_430-447_). Therefore, we suspected that alternative sequences were targeted, and performed a detailed epitope mapping of 21OH_430-447_.

PBMCs from patients and controls were stimulated with a panel of overlapping 9-mer peptides (Pep1-10) covering Pep34 (21OH_430-447_), as well as Pep34 itself, and were assayed for IFNγ secretion using ELISPOT (**Figure 3**). Five out of ten tested patients displayed responses above background/DMSO to Pep34, and among these, four had a clear response to Pep5 (peptide sequence: ARLELFVVL, 21OH_434-442,_ **Figure 3**). Responses to the other peptides were generally low.

In order to test whether ARLELFVVL could represent an alternative HLA-B8 restricted epitope, we took advantage of the REVEAL epitope discovery system offered by ProImmune, in which 8-mer and 9-mer peptides covering Pep34 were synthesized and tested for their ability to bind HLA-B*0801 (**Supplementary Tables 8**–**11**). Individual binding scores (REVEAL scores) were calculated as percentage binding compared to a positive control peptide. Based on a threshold of ≥45% of the control, ARLELFVVL did not show any affinity to HLA-B*0801 (REVEAL score = 0.3), in contrast to EPLARLEL that was considered to be a strong ligand (REVEAL score = 78).

### T Cell Responses to ARLELFVVL (21OH_434-442_) Are Predominantly Restricted by HLA*C0701

As we had established that ARELELVVL could not be a HLA-B*0801 ligand, we started to search for alternative HLA class I molecules able to present this peptide. Initially, we used the NetMHCpan-4.1 server that predicts the binding of peptides to specified HLA molecules. We had a particular emphasis on the ancestral haplotype 8.1 (AH 8.1) as the HLA class I alleles (HLA-A*01 and HLA-C*0701) within AH 8.1 is in strong linkage disequilibrium (LD) with the predisposing HLA-B8, -DR3 and -DQ2 alleles. HLA-A*01 was not predicted to bind ARLELFVVL, or to any possible 8-, 9-, 10- or 11-mer peptide from Pep34 (21OH_430-447_). In contrast, HLA-C*0701 was predicted to bind ARLELFVVL, but not any other possible 8-, 9-, 10- or 11-mer peptide from Pep34. We also assessed to what degree other HLA class I molecules not part of the AH 8.1 were predicted to bind ARLELFVVL (**Supplementary Table 12**). Several other HLA class I molecules were predicted to bind ARLELFVVL, but these were all rare among AAD patients, with allele frequencies ranging from 0.0% to 5.7%. The only exception was HLA-C*0702, present in 14.5% of AAD patients, which only differs from HLA-C*0701 by two amino acids and has a very similar peptide binding pocket. Notably, HLA-C*0701 is the most common HLA-C allele among Norwegian AAD patients, being present in 33.4% of patients but only 14.7% of controls (10).

As the crystal structure of HLA-C*0702 has been solved, we evaluated the electrostatic potential of its peptide binding cleft, and modeled potential interactions of ARLELFVVL and HLA-C*0701 by use of the PyMOL software (**Supplementary Figure S4**). The electrostatic potential of the B pocket of HLA-C*0701 protein, which probably contains an anchor site of peptide ligands, displayed an excess of negative charges at the surface, indicating a potential to bind positively charged peptide residues (S4A). Indeed, the molecular models indicated that the positively charged arginine residue at position 2 of ARLELFVVL may serve to anchor the peptide to the floor of the antigen-binding cleft of HLA-C*0701 *via* a salt bridge with aspartic acid at amino acid position 9 (p.Asp9) and a hydrogen bond with serine at amino acid position 24 (p.Ser24) of the HLA molecule (S4B). At the same time, hydrophobic interactions between leucine 9 of the peptide and leucines at positions 81 and 95 of HLA-C*0701 could potentially ensure the stabilization of the peptide-HLA interaction further (S4C).

To confirm the relationship between HLA-C*0701 and ARLELFVVL (21OH_434-442_), we purchased custom-designed C7*ARLELFVVL streptamers to determine the frequency of ARLELFVVL-specific CD8^+^ T cells among PBMCs isolated from 13 patients (P18-20, P23, P25-P30, P32-P34). *Ex vivo* staining of PBMCs with streptamers revealed populations of ARLELFVVL-specific CD8^+^ T cells in >70% of the patients tested (**Figure 4A**). The highest individual frequency observed was 0.32% of total CD8^+^ T cells. The overall frequency of ARLELFVVL-specific cells was significantly higher in patients than controls (*P* = 0.0021).

Subsequently, we stimulated cells from six of the patients and five of the controls with the ARLELFVVL peptide and attempted to expand the cells for 13 days under similar conditions as for LLNATIAEV and EPLARLEL. A marked expansion was evident in all patients, including some with low levels of ARLELFVVL-specific cells on day 0 (**Figure 4B**), with frequencies of C7*ARLELFVVL streptamer-positive cells reaching as high as 1.5% of CD8^+^ T cells on day 13 (**Figure 4C**). In contrast, no signs of expansion were seen for any the controls.

Having demonstrated the presence of circulating ARLELFVVL-specific CD8^+^ T cells in AAD patients, we also assessed their ability to secrete IFNγ by ELISPOT. PBMCs from 14 patients (P18-P20, P23-P33) and 14 healthy controls were stimulated with ARLELFVVL and Pep34 for 40h. Together, the patients had significantly higher IFNγ responses to ARLELFVVL after background subtractions than the controls (*P <*0.0001, **Figure 4D**). This was also the case for the longer peptide, Pep34 (*P <*0.0001, **Figure 4D**). Notably, the strongest responding patients had comparable reactivity against Pep34. In contrast, none of the controls displayed IFNγ responses above the background (except for one control with just a single IFNγ SFC).

### Several Microbes Known to Colonize Humans Contain Homologous Sequences to LLNATIAEV (21OH_342-350_) and ARLELFVVL (21OH_434-442_)

BLAST searches revealed high sequence homology between proteins from several human pathogens and both 21OH-derived T cell epitopes investigated in this study. Peptide hits were predominantly from bacteria (both facultative and obligate intracellular, as well as extracellular species) but also a few parasitic helminths. The microbial species and the corresponding proteins containing highest sequence homology are enlisted in **Tables 1** and **2**.

**Table 1:** Peptides derived from intracellular bacteria reported to colonize humans with closest sequence homology to the CD8+ T cell restricted epitopes of 21OH.

Organism	Predicted HLA-C7 ligand	Accession nr, Protein, amino acids	BLAST bit score	Predicted HLA-A2 ligand	Protein, amino acids	BLAST bit score
***Homo sapiens***	ARLELFVVL	P08686.1, Steroid 21-hydroxylase, 434-442	31.2	LLNATIAEV	P08686.1, Steroid 21-hydroxylase, 342-350	30.3
**Intracellular bacteria (**obligate or facultative**)**						
*Mycobacterium intracellulare*	ARLELHVML	WP_095763105.1, Cytochrome P450, 376-384	21.4	LLGAVIAEV	WP_008262926.1, IS21 family transposase, 63-71	21.4
*Mycobacterium avium*	ARMELRVVL	WP_019729975.1, Cytochrome P450, 344-352	21	LLAAVIAEV	WP_151834795.1, IS21 family transposase, 65-73	21
*Mycobacterium kansasii*	ARLELRVML	KZS57495.1, Cytochrome, 377-385	20.2	LLNATITQV	KZS66019.1, penicillin-binding protein, 20-28	24.4
*Mycobacterium tubercolosis*	ARLELPVIL	WP_152318453.1, ROK family protein, partial, 16-24	21	LLNTSISEV	WP_220689542.1, NAD-binding protein, partial, 76-84	21.8
*Legionella pneumophila*	ARLEMHVVL	HAU3761196.1, cytochrome P450, 337-345	22.3	LVNPTIAEV	WP_061484691.1, Cytochrome P450, 142-150	23.1
*Salmonella enterica*	ARLELFLLV	EGF9691577.1, metal ABC transporter permease, 179-187	23.1	LLNAIIAEI	WP_136057839.1, P-type conjugative transfer ATPase TrbB, 163-171	24.4

**Table 2:** Peptides derived from extracellular bacteria and helminths reported to colonize humans with closest sequence homology to the CD8+ T cell restricted epitopes of 21OH.

Organism	Predicted HLA-C7 ligand	Accession nr, Protein, amino acids	BLAST bit score	Predicted HLA-A2 ligand	Protein, amino acids	BLAST bit score
***Homo sapiens***	ARLELFVVL	P08686.1, Steroid 21-hydroxylase, 434-442	31.2	LLNATIAEV	P08686.1, Steroid 21-hydroxylase, 342-350	30.3
**Extracellular bacteria**						
*Pseudomonas alcaligenes*	ARLELFAVL	MBB4822134.1, type VI secretion system protein ImpJ, 437-445	27.4	LLGATIPEV	WP_187803995.1, acyl-CoA dehydrogenase, 64-72	22.3
*Stenotrophomonas maltophilia*	ARLEQFAVL	VUJ40478.1, Miniconductance mechanosensitive channel YbdG, 290-298	22.3	LLNAAIAGV	WP_049466125.1, EAL domain-containing protein, 129-137	21.8
*Actinomyces naeslundii*	ARLAMFIVL	WP_076066049.1, DUF998 domain-containing protein, 15-23	22.3	LLAASIAEV	WP_004564635.1, type II secretion system F family protein, 69-77	22.3
*Vibrio parahaemolyticus*	ARLELAIVL	HAS6787063.1, phosphatase, 202-210	21.8	LLNETIAEV	WP_065771913.1, sensor histidine kinase, 430-438	26.9
*Shewanella putrefaciens*	ARFELFIVL	WP_113939870.1, DUF2339 domain-containing protein, 664-672	24.8	LIDATIVEV	WP_006080724.1, GatB/YqeY domain-containing protein, 102-110	21
**Helminths (nematodes)**						
*Toxocara canis*	ARMELFIIL	KHN85744.1, Cytochrome P450 2C25, 482-490	24.4	LLNETISEV	KHN84767.1, Phosphorylase b kinase gamma catalytic chain, liver/testis isoform, partial, 108-116	24.4
*Anisakis simplex*	ARMELFIIL	VDK19997.1, unnamed protein product, 62-70	24.4	LLNETISEV	VDK48986.1, unnamed protein product, 58-66	24.4

## Discussion

We report a novel CD8^+^ T cell restricted immunodominant epitope of 21OH, the major autoantigen of the adrenal cortex in AAD. This epitope, ARLELFVVL (21OH_434-442_), is presented by HLA-C7 and is the first HLA-C7 restricted epitope to be reported for an autoimmune disease. Two other dominant epitopes have previously been proposed: EPLARLEL (21OH_431-438_), restricted by HLA-B8 (12), and LLNATIAEV (21OH_342-350_) restricted by HLA-A2 (13). Using peptide-loaded HLA dextramers, we could readily detect LLNATIAEV-specific CD8^+^ T cells in PBMCs from patients with AAD, but could not confirm presence of significant numbers of EPLARLEL-specific CD8^+^ T cells. Instead, we identified ARLELFVVL as a second immunodominant epitope presented by HLA-C7.

Initially, we investigated T cell responses against the previously reported HLA-A2 and HLA-B8-restricted epitopes. EPLARLEL-specific CD8^+^ T cells appeared to be less frequent and proved more difficult to expand *in vitro* than LLNATIAEV-specific cells. This was not due to experimental conditions such as the use of cryopreserved PBMC, as comparing fresh and frozen cells from patients did not reveal any differences. Similar to our findings, Dawoodji et al. showed only modest expansion of EPLARLEL-specific cells (13). The lack of convincing data on EPLARLEL-specific T cells, in spite of robust IFNγ production in response to longer C-terminal peptides including EPLARLEL by both Dawoodji et al. and Rottembourg et al. spurred us on to do a more comprehensive investigation. Using short, overlapping peptides derived from a longer C-terminal peptide, Pep34, ARLELFVVL (21OH_434-442_) stood out as the predominant target for patient-specific T cell responses against Pep34. However, the REVEAL assay carried out by Proimmune confirmed that this peptide was not a likely binder of HLA-B8. We therefore wondered if ARLELFVVL could be restricted to a different HLA class I allele.

The ancestral haplotype that contains HLA-B8 alleles is termed 8.1, and its extended form is usually defined by the haplotype HLA-A*01:01, -C*07:01, -B*08:01, -DRB1*03:01, -DQA1*05:01 and -DQB1*02:01, which together have been highly conserved throughout evolution (23). HLA-B8 is therefore in strong LD with the other class I alleles HLA-C*0701 and HLA-A*0101. Using the NetMHC 4.0 server for HLA peptide binding prediction, ARLELFVVL was predicted as a potential ligand of HLA-C*0701. Based on this observation, C7*ARLELFVVL streptamers were generated to determine the frequency of ARLELFVVL-specific CD8^+^ T cells among PBMCs from 13 patients. Intriguingly, ARLELFVVL-specific cells were readily detected in the majority of the patients, but were much less frequent in the controls. These cells also displayed the ability of clonal expansion in all the patients tested (but not the controls), when stimulated with ARLELFVVL and cultured in the presence of IL-7 and IL-2 for 13 days *in vitro*. Notably, omitting IL-7 resulted in high cell death and very little clonal expansion, underlining the need of IL-7 for T cell survival and homeostasis in such cultures (16, 17).

Consistent with these findings, we also detected IFNγ responses in the same patients with demonstrated ARLELFVVL-specific CD8^+^ T cells. At least 10 out of 14 patients, but none of the controls, were found to have detectable IFNγ production against ARLELFVVL *ex vivo*. The same patients (except for P26, and P29) had comparable responses to Pep34 (21OH_430-447_), suggesting that the responses against the longer peptide are primarily directed against ARLELFVVL, and not EPLARLEL as previously proposed.

Studies on the HLA-C locus have been rather few compared to HLA-A and HLA-B, especially when it comes to autoimmunity and self-reactive T cells, with cross-reactive CD8^+^ T cells from patients with psoriasis targeting peptides of both keratin 17 and streptococcal M-proteins in the context of HLA-C*0602 as a notable exception (24, 25). HLA-C is expressed at lower levels than HLA-A/B on the cell surface and plays an important role as a ligand for killer cell immunoglobulin-like receptors (KIR), expressed by natural killer (NK) cells (26). KIRs and HLA-C interact to regulate the cytotoxic activity of NK cells, ensuring balance between immune responses and self-tolerance (27–29). Polymorphisms of KIRs and HLA may influence this balance in subtle ways, potentially increasing the risk of developing autoimmune diseases or unfavorable outcomes to infections and transplantations. With regards to AAD, one study has suggested a decreased function of NK cells, but it is unclear whether this trait is genetically determined (30).

HLA-C7 as an antigen-presenting molecule within the context of autoimmunity has received little attention. This may be due to the strong LD within the 8.1 haplotype region that makes it difficult to detect any independent associations for individual HLA variant loci (23). Interestingly, it was recently reported that the antigen binding cleft of HLA-C*0702 (and by molecular similarity also HLA-C*0701) is rather narrow and deep, which severely limits its peptide-binding repertoire (22). The narrow peptide-binding cleft may contribute to low cell surface expression of HLA-C*0701, as the binding of peptides *per se* serve as a post-translational regulatory mechanism. This raises the question of whether ARLELFVVL, being restricted to the narrow cleft of HLA-C*0701, promotes the expression of HLA-C*0701 and stabilizes it on the cell surface at a higher level than on other tissues. In the adrenals, where 21OH-derived peptides probably are abundant, this could be a relevant mechanism by which adrenocortical cells are targeted for destruction by CD8^+^ T cells as ARLELFVVL may increase the stability of HLA-C*0701 cell surface expression.

The majority of AAD patients carrying either the HLA-A2 or HLA-C7 alleles demonstrated T cell responses against LLNATIAEV or ARLELFVVL, respectively. The *ex vivo* frequencies of LLNATIAEV- and ARLELFVVL-specific CD8^+^ T cells in patients with AAD are comparable to self-antigen specific CD8^+^ T cell responses associated with T1D (31, 32). Also similar to T1D related self-specific T cells, LLNATIAEV- and ARLELFVVL-specific T cells were observed in some control samples, although at significant lower numbers than for the patients. Furthermore, LLNATIAEV- and ARLELFVVL-specific cells from patients displayed an increased ability of clonal expansion compared to controls following stimulation with cognate peptide, IL-7 and IL-2 supplementation, which could possibly reflect a phenotypic difference. For instance, T cells specific for T1D-associated self-antigens glutamic acid decarboxylase- (GAD) 65 and preproinsulin (PPI) are frequent in peripheral blood from both healthy individuals and patients, but are predominantly naïve in healthy and skewed towards a memory phenotype in the patients, indicating antigen-driven differentiation in an inflammatory setting in the latter (31–33). Antigen experience *in vivo* may also explain the apparent expansion of LLNATIAEV- and ARLELFVVL-specific T cells in the blood of AAD patients.

Curiously, we observed some expansion of LLNATIAEV-specific cells with numerous IFNγ SFCs upon restimulation in one control subject. However, net cytokine responses were low compared to the patients and IFNγ SFCs were only detected following expansion (in contrast to patients), which may suggest a different phenotype of these cells. The control subject was negative for 21OH autoantibodies, making unrecognized AAD or pre-clinical AAD unlikely as such antibodies are present in 85% of patients (2, 3) and often precede the development of clinical disease (4). It should be noted that we used a relatively high concentration of peptide in our experiments, which potentially can provide sufficient stimuli to allow for T cell expansion in the presence of otherwise inadequate levels of co-stimulation (34). Although we cannot rule out that the propensity of LLNATIAEV-specific cells in the control subject to expand could be of pathological significance, additional factors such as genetic predisposition and environmental agents may have to be in place for these cells to become harmful. The lack of 21OH autoantibodies in this individual could indicate that such factors are not present. Given the relatively small number of controls used for the LLNATIAEV stimulation experiments, it is difficult to estimate how common this phenomenon could be in healthy controls. Still, Dawoodji et al. tested a larger number of controls against the longer peptide 21OH_337-354_ without detecting any significant IFNγ responses (13).

Apart from IFNγ, cytokine screens indicated variable production of pro-inflammatory cytokines IL-6, TNFα, MIP-1α and MIP-1β on day 0 in response to LLNATIAEV, suggesting a pro-inflammatory phenotype in the patients. Similar cytokine patterns have been found for PPI-specific CD8^+^ T cell clones from T1D patients (32, 35). Concerning the pathogenesis of AAD, these cytokines may have direct impact on the function and viability of adrenocortical cells as has been reported for IL-6 and TNFα (36, 37). Based on the preliminary nature of these results, the cytokine production of 21OH-specific T cells must be investigated more comprehensively in future studies in order to make reasonable conclusions. Still, for the detection of 21OH-specific T cell activation, IFNγ seems to be a reliable and robust biomarker.

Finally, although we hypothesize that the activation of autoreactive 21OH-specific T cells *in vivo* is a critical step in the development of AAD, the mechanisms behind the formation of such potentially pathogenic T cells is unknown. A popular concept in autoimmunity is so-called molecular mimicry, which may occur when similarities between foreign and self-peptides favor activation of autoreactive T cells (38). By use of a BLAST-based approach searching microbial proteomes *in silico* for peptide sequences with high homology to LLNATIEV and ARLELFVVL, we identified a number of microbial peptides from human pathogens, predominantly bacteria. Bacterial species containing proteins with high sequence homology to the 21OH-derived epitopes included both common pathogens widely distributed in the environment such as nontuberculous mycobacteria species and *Salmonella enteridis*, but also pathogens rarely causing disease in Norway, such as *Actinomyces naeslundi* and the polycyclic aromatic hydrocarbons-consuming bacteria *Pseudomonas alcaligenes*. Interestingly, homologous sequences in a couple of parasitic helminths, the nematodes *Anisakis simplex*, common in saltwater fish in Norwegian waters, and *Toxocara canis*, occurring in Norwegian pets, were also identified. Follow-up studies are needed to clarify any potential role for these microbial species in precipitating the development and activation of 21OH-specific CD8+ T cells in AAD patients.

Based on our findings, we conclude that 21OH-specific CD8^+^ T cells in AAD patients are predominantly restricted by HLA-A2 and HLA-C7 molecules, directed against the LLNATIAEV and ARLELFVVL peptide epitopes, respectively. We believe that the definition of these CD8^+^ T cell restricted epitopes will prove immensely valuable in future studies of the pathogenesis of AAD, and potentially also for clinical studies involving immunotherapy. Specifically, the use of HLA dextramers and streptamers as we have proven suitable for AAD patients, should allow for deep phenotyping and functional scrutiny of self-reactive T cells at the single-cell level.

## Data Availability Statement

The original contributions presented in the study are included in the article/**Supplementary Material**. Further inquiries can be directed to the corresponding author.

## Ethics Statement

The studies involving human participants were reviewed and approved by REC West - Secretariat: the Regional Committee for Medical and Health Research Ethics. The patients/participants provided their written informed consent to participate in this study.

## Author Contributions

AH, SA, and EB designed experiments and figures and wrote the manuscript. AH, SA, KE, KB, and EB conducted experiments. EH, LB, and Ø.B, compiled clinical data and information. ER and AW collected genetic data. All authors contributed to the article and approved the submitted version.

## Funding

This study was funded by the KG Jebsen Foundation, the Research Council of Norway (grant number 262677), the Novo Nordisk Foundation (grant number NNF14OC0011005) and the Health authorities of Western Norway.

## Conflict of Interest

The authors declare that the research was conducted in the absence of any commercial or financial relationships that could be construed as a potential conflict of interest.

## Publisher’s Note

All claims expressed in this article are solely those of the authors and do not necessarily represent those of their affiliated organizations, or those of the publisher, the editors and the reviewers. Any product that may be evaluated in this article, or claim that may be made by its manufacturer, is not guaranteed or endorsed by the publisher.
