# Supplementary material for: 21-Hydroxylase-Specific CD8+ T Cells in Autoimmune Addison’s Disease Are Restricted by HLA-A2 and HLA-C7 Molecules
Source: Front Immunol. 2021 Oct 14;12:742848. doi: 10.3389/fimmu.2021.742848 (PMC8551825; doi:10.3389/fimmu.2021.742848)
Supplement: Supplementary file 1 [file DataSheet_1.pdf]

Table of Contents

Supplementary Figures

Supplementary Figure 1. Flow cytometry gating strategy

Supplementary Figure 2. *Ex vivo* dextramer plots from carriers of both HLA-A2 and HLA-B8

Supplementary Figure 3. Pro-inflammatory cytokine responses to LLNATIAEV

Supplementary Figure 4. Molecular models of potential interactions between HLA-C*0701 and ARLELFVVL

Supplementary Tables

Supplementary Table 1. Patient characteristics

Supplementary Tables 2-7. Overview of multiplex cytokine data from peptide stimulations

Supplementary Table 8. Peptide information for REVEAL® MHC peptide binding assay (Module 1)

Supplementary Table 9. B*08:01 Binding Data from REVEAL® MHC peptide binding assay (Module 2)

Supplementary Table 10. ProVE® Pentamer Library Analysis from REVEAL® MHC peptide binding assay (Module 3)

Supplementary Table 11. Summary of the binding and rate data for peptides passing the ProImmune REVEAL® MHC peptide binding assay

Supplementary Table 12. Additional HLA class I molecules predicted to bind ARLELFVVL and their frequencies in patients and controls

Accompanying tables containing source data for figures

Accompanying table to Figure 1. Dextramer frequencies expressed as % of total CD8+ T cells

Accompanying table to Figure 2. ELISPOT results expressed as IFNγ SFC per 6 x 10^5 PBMC (Blank subtracted)

Accompanying table to Figure 3. ELISPOT results expressed as IFNγ SFC per 6 x 10^5 PBMC (Blank subtracted)

Accompanying table to Figure 4. Streptamer frequencies expressed as % of total CD8+ T cells and ELISPOT results expressed as IFNγ SFC per 6 x 10^5 PBMC (Blank subtracted)

Supplementary Figure 1. Flow cytometry gating strategy

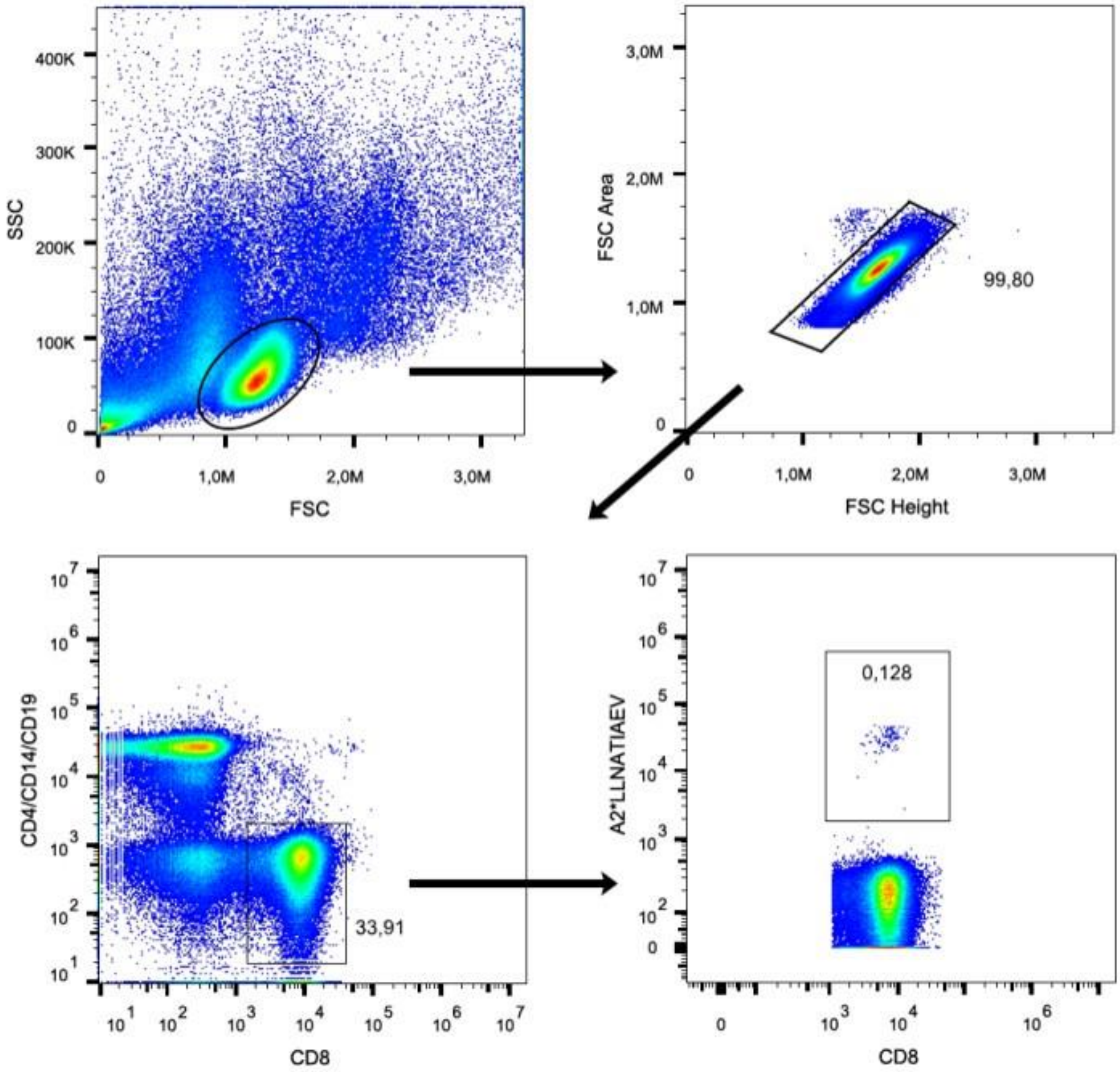

Supplementary Figure 1. The figure illustrates the sequential gating strategy for quantification of dextramer-positive CD8+ T cells. Initial lymphocyte gates (top left) were subjected to doublet exclusion (top right) and then gated on CD8+ T cells while excluding CD4+ (T helper cells), CD14+ (monocytes) and CD19+ (B cells) cells (bottom left). A conservative dextramer gate was drawn to reduce false positives and remained fixed for all the analyses. Gate numbers represent percentages of the parent population.

Supplementary Figure 2. *Ex vivo* dextramer plots from carriers of both HLA-A2 and HLA-B8

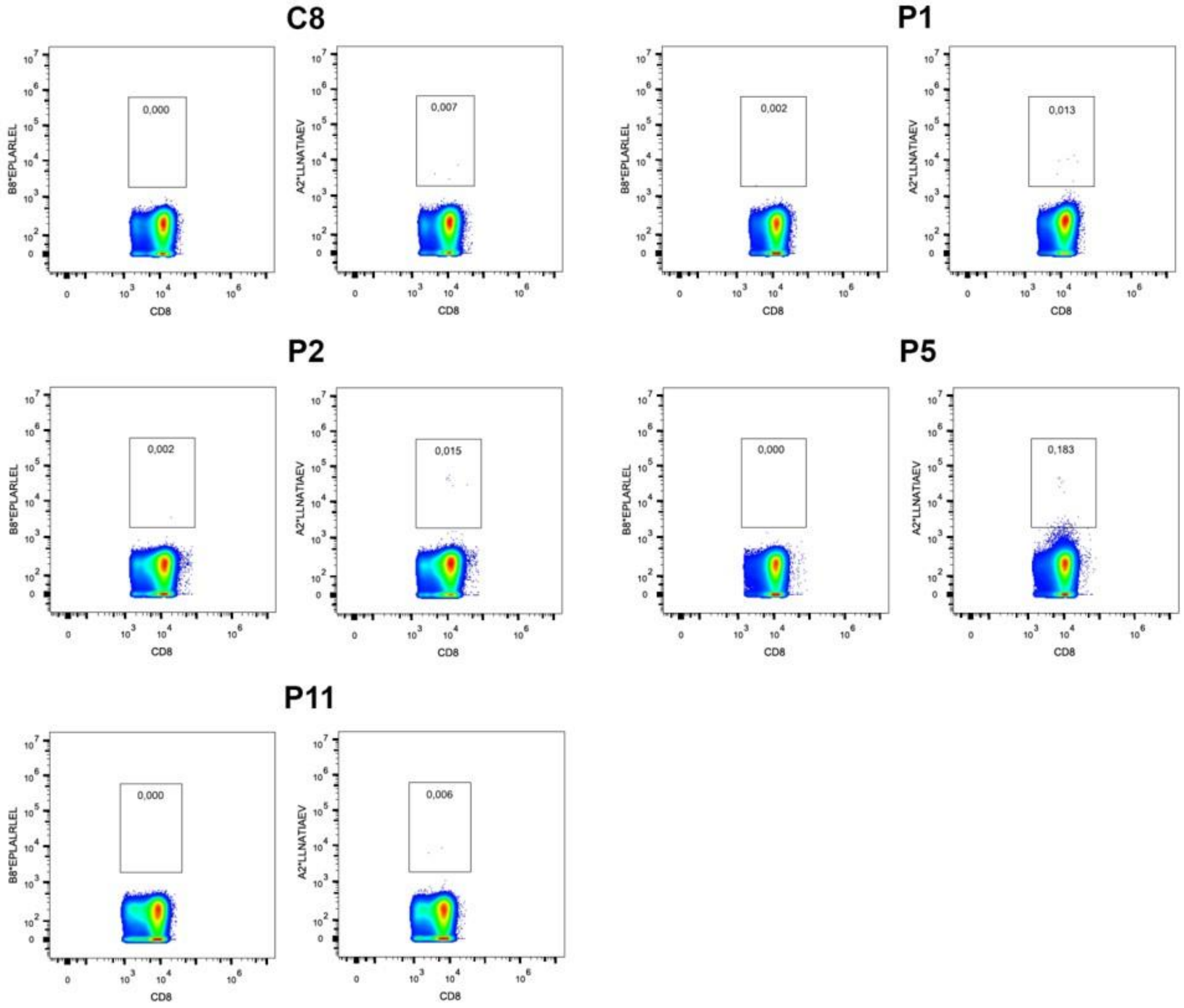

Supplementary Figure 2. The figure shows *ex vivo* stainings with A2*LLNATIAEV and B8*EPLARLEL dextramers for individuals co-expressing HLA-A2 and HLA-B8. The dextramer frequencies in this experiment were used to determine which peptide to use in subsequent stimulation experiments (except for P11 where both peptides were used).

Supplementary Figure 3. Pro-inflammatory cytokine responses to LLNATIAEV

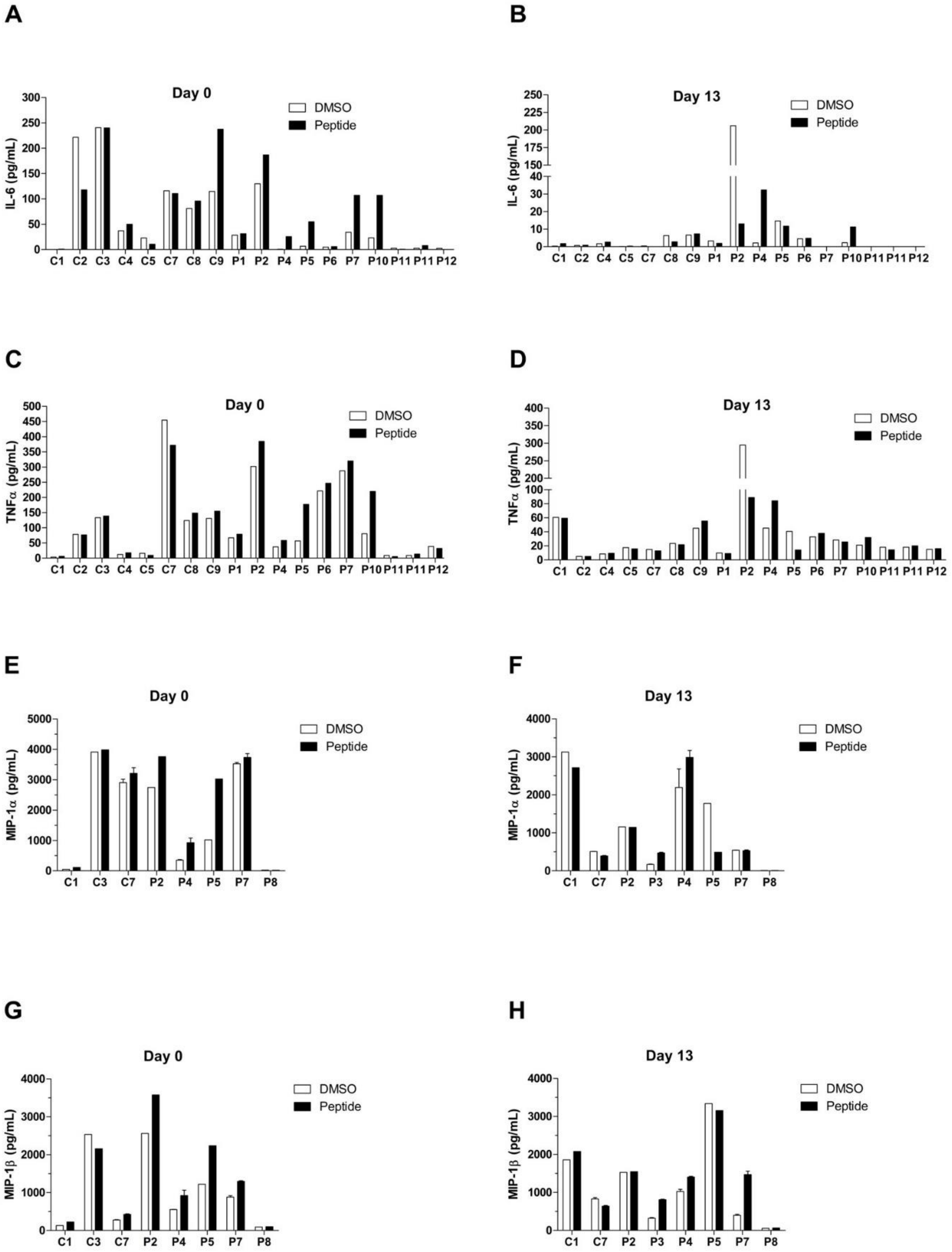

Supplementary Figure 3. Supernatants from ELISPOT assays were analyzed for individual cytokines by ELISA (**A-D**) or a panel of 17 cytokines by multiplex technology (**E-H**). Shown in the figure are the results for IL-6 (**A, B**), TNFα (**C, D**), MIP-1α (**E, F**) and MIP-1β (**G, H**)

upon ex vivo stimulation (**A, C, E, G**) and restimulation after in vitro expansion (**B, D, F, H**). Results from ELISAs are expressed as means of duplicates, while the results from multiplex analyses are either means of duplicates or single well measurements.

Supplementary Figure 4. Molecular models of potential interactions between HLA-C*0701 and ARLELFVVL

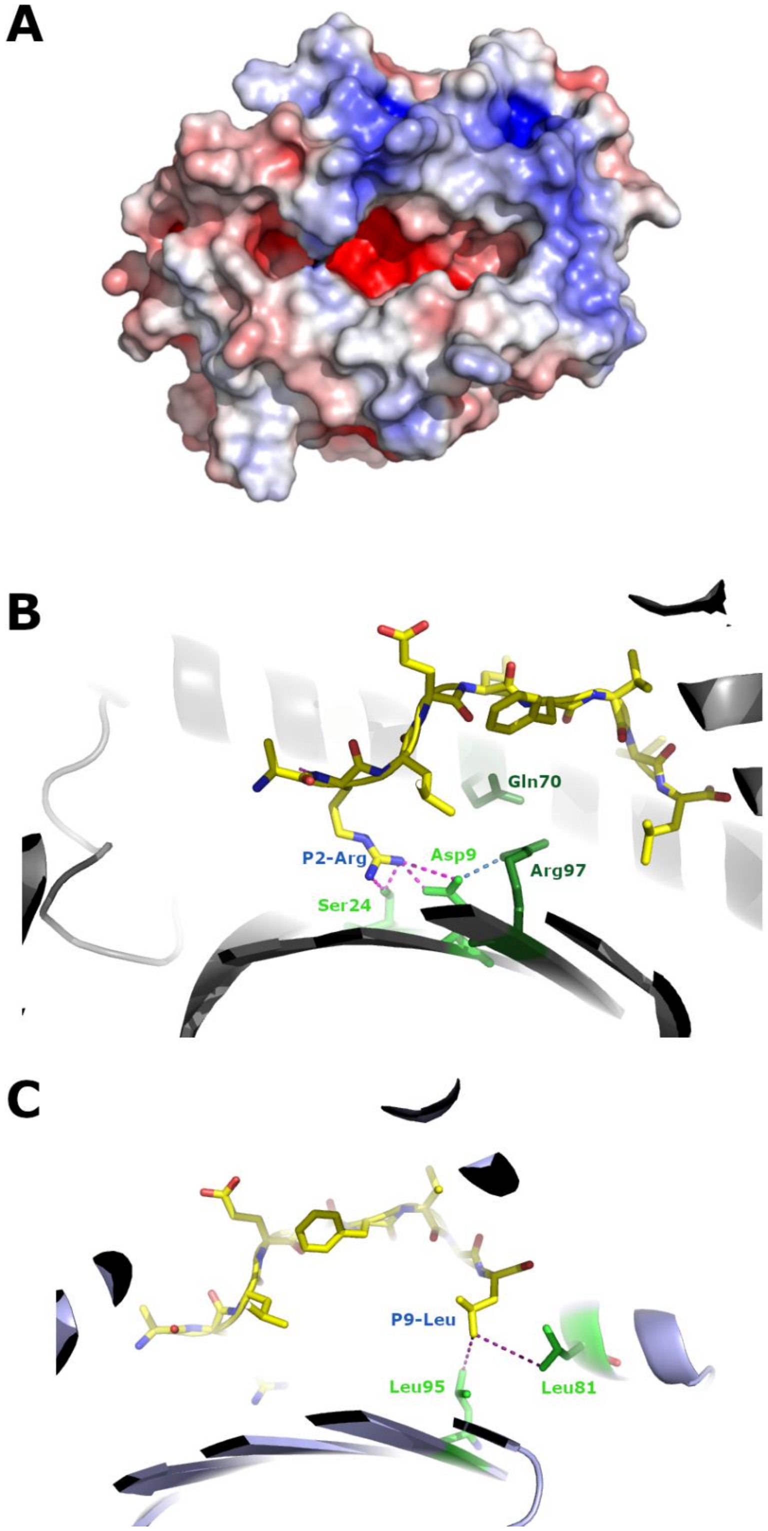

Figure 4. Molecular models of potential interactions between HLA-C*0701 and ARLELFVVL. **(A)** Visualization of the electrostatic potential of the peptide pending cleft of HLA-C*0702. Positively charged electrostatic potential is shown in blue color, negatively charged in red. More intense colors indicate stronger electrostatic potential. **(B)** Arginine at position 2 of the ARLELFVVL serves to anchor the peptide to the floor of the antigen-binding cleft of HLA-C*0701 via a salt bridge with aspartic acid at amino acid position 9 (Asp9) and a hydrogen bond with serine at amino acid position 24 (Ser24) of the HLA molecule. Arg70 and possibly also Gln70 may contribute to the anchor site. **(C)** Hydrophobic interactions between leucine 9 of ARLELFVVL and leucines at positions 81 and 95 of HLA-C*0701 may potentially serve to stabilize the peptide-HLA interaction further.

Supplementary Table 1. Patient characteristics

Patient ID	Sex	Other Autoimmune Diseases	Disease Duration at Sampling (y)	Age at Diagnosis (y)	21OH Ab Index[1]	HLA-A		HLA-B		HLA-C		HLA-DRB1		HLA-DQA1		HLA-DQB1		HLA-DPB1	
P1	M	T1D	52	11	468	02:01	24:02:00	08:01	40:01:00	03:04	07:01	04:01	08:01	03:01	04:01	03:02	04:02	NA	
P2	F	Hypothyroidism	31	19	236	01:01	02:01	08:01	40:01:00	03:04	07:01	03:01	04:04	03:01	05:01	02:01	03:02	04:01	04:01
P3	M	Hyper, Alopecia, Vitiligo	16	24	971	02:01	11:01	08:01	15:01	03:04	07:01	03:01	04:01	03:01	05:01	02:01	03:02	04:01	04:01
P4	M	Hypothyroidism	7	18	961	01:01	11:01	07:02	08:01	07:01	07:02	03:01	15:01	01:02	05:01	02:01	06:02	01:01	04:01
P5	M	Vitiligo	9	13	424	01:01	02:01	08:01	15:01	03:04	07:01	03:01	04:01	03:01	05:01	02:01	03:02	01:01	04:01
P6	F	Type 1 diabetes	0	29	1175	01:01	31:01:00	08:01	15:01	03:04	07:01	03:01	04:01	03:01	05:01	02:01	03:02	NA	
P7	F	None	5	23	848	02:01	11:01	07:02	27:05:00	02:02	07:02	12:01	13:01	01:03	05:05	03:01	06:03	NA	
P8	F	None	0	45	1374	01:01	24:02:00	08:01	15:01	03:03	07:01	03:01	04:01	03:01	05:01	02:01	03:02	02:01	04:01
P9	F	Type 1 diabetes	4	34	583	02:01	31:01:00	15:01	40:01:00	03:04	03:04	04:01	04:04	03:01	03:01	03:02	03:02	04:01	04:02
P10	M	Vitiligo	4	22	557	02:01	02:01	40:01:00	47:01:00	03:04	06:02	03:01	04:01	03:01	05:01	02:01	03:02	01:01	04:01
P11	M	APS-1	28	10	488	02		08		NA		03:01	13:01	NA		02:01	06:03	NA	
P12	F	None	3	25	822	01:01	25:01:00	08:01	15:01	03:04	07:01	03:01	04:01	03:01	05:01	02:01	03:02	04:01	04:01
P13	F	Type 1 diabetes, Hypothyroidism	18	40	742	01:01	03:01	08:01	08:01	07:01	07:01	03:01	03:01	05:01	05:01	02:01	02:01	02:01	04:01
P14	M	Type 1 diabetes, Hypothyroidism	7	13	1101	01:01	24:02:00	08:01	08:01	07:01	07:01	03:01	04:01	03:01	05:01	02:01	03:02	04:01	04:01
P15	F	Type 1 diabetes, Hypothyroidism	21	36	662	01:01	11:01	08:01	08:01	07:01	07:01	03:01	04:04	03:01	05:01	02:01	03:02	04:01	04:01
P16	F	Hypothyroidism, Pernicious anemia, Vitiligo	7	33	841	01:01	24:02:00	08:01	40:01:00	03:04	07:01	03:01	04:04	03:01	05:01	02:01	03:02	02:01	04:02
P17	F	Celiac disease, Primary ovarian insufficiency	36	33	778	01:01	01:01	08:01	08:01	07:01	07:01	03:01	15:01	01:02	05:01	02:01	06:02	02:01	04:01
P18	M	Hypothyroidism	9[2]<br>10[3]	22	505[2]<br>414[3]	01:01	03:01	07:02	08:01	07:01	07:02	03:01	15:01	01:02	05:01	02:01	06:02	02:01	04:01
P19	F	Hyperthyroidism, Vitiligo	3[2]<br>1[3]<br>3[4]	18	584[2]<br>1160[3]<br>595[4]	01:01	24:02:00	08:01	08:01	07:01	07:01	03:01	04:01	03:01	05:01	02:01	03:02	04:01	04:01
P20	F	Hypothyroidism	4[2]<br>5[3]	25	748[2]<br>752[3]	01:01	33:01:00	08:01	14:02	07:01	08:02	01:02	03:01	01:01	05:01	02:01	05:01	01:01	02:01
P21	M	Hypothyroidism	27	18	575	25:01:00	32:01:00	08:01	40:01:00	03:04	07:01	03:01	04:04	03:01	05:01	02:01	03:02	NA	
P22	F	Hypothyroidism	23	28	378	01:01	03:01	08:01	27:05:00	01:02	07:01	03:01	04:04	03:01	05:01	02:01	03:02	01:01	04:01
P23	F	Hypothyroidism	5	37	283	01:01	02:01	08:01	44:02	05:01	07:01	03:01	04:01	03:03	05:01	02:01	03:01	04:01	04:01
P24	F	None	18	21	600	01:01	24:02	08:01	27:05	02:02	07:01	03:01	04:01	03:01	05:01	02:01	03:02	04:01	04:01
P25	F	Hypothyroidism	0	58	1273	01:01	02:01	08:01	44:02	05:01	07:01	03:01	04:01	03:03	05:01	02:01	03:01	NA	
P26	F	None	4	26	527	01:01	03:01	08:01	40:01	03:04	07:01	03:01	04:04	03:01	05:01	02:01	03:02	NA	
P27	F	T1D, Hypothyroidism, Hypoparathyroidism	0	41	601	NA		NA		07:01		NA		NA		NA		NA	

P28	F	Hypothyroidism	7	57	500	01:01	24:02	08:01	35:03	04:01	07:01	03:01	12:01	05:01	05:05	02:01	03:01	01:01	04:01
P29	M	Hyperthyroidism	1	20	515	01:01	33:01	08:01	08:01	07:01	07:01	03:01	03:01	05:01	05:01	02:01	02:01	01:01	04:01
P30	F	Hypothyroidism	2	40	572	01:01	02:01	07:02	08:01	07:01	07:02	03:01	04:01	03:01	05:01	02:01	03:02	04:01	04:01
P31	F	T1D, Hyperthyroidism, Vitiligo	7	47	831	01:01	03:01	08:01	44:02	05:01	07:01	03:01	04:01	03:01	05:01	02:01	03:02	NA	
P32	M	Vitiligo	11	14	351	01:01	02:01	08:01	15:01	03:04	07:01	03:01	04:01	03:01	05:01	02:01	03:02	01:01	04:01
P33	M	Hypothyroidism	25<br>27[4]	15	803<br>844[4]	01:01	02:01	08:01	15:01	03:04	07:01	03:01	04:01	03:01	05:01	02:01	03:02	04:01	04:01
P34	F	Primary Ovarian Insufficiency, Vitiligo	14<br>15[4]	35	726<br>650[4]	01:01	33:03	08:01	51:01	07:01	15:02	03:01	04:07	03:03	05:01	02:01	03:01	03:01	04:01

[1] In-house radioimmunoassay used for establishing disease etiology; antibody (Ab) index ≥ 56 denotes positivity.
[2] Sample used for experiments with LLNATIAEV and EPLARLEL.
[3] Sample used for experiments with ARLELFVVL.
[4] Sample used for expansion upon ARLELFVVL stimulation.

Supplementary Table 2. Overview of multiplex cytokine data (GM-CSF, sCD137 and IFNγ, quantified as pg/ml) from peptide stimulations.

ID	Day	Stimulant	GM-CSF	SD	sCD137	SD	IFNγ	SD
**C1**	0	Blank	0,8	-	#NUM	-	#NUM	-
**C1**	0	LLNATIAEV	1,5	-	#NUM	-	0,5	-
**C1**	13	Blank	136,6	-	20,9	-	7,3	-
**C1**	13	LLNATIAEV	223,3	-	26,7	-	3,1	-
**C3**	0	Blank	10,4	-	1,6	-	1,6	-
**C3**	0	LLNATIAEV	11,4	-	0,3	-	3,1	-
**C7**	0	Blank	5,2	0,2	4,7	1,2	0,0	0,0
**C7**	0	LLNATIAEV	5,2	0,2	5,5	0,0	0,0	0,0
**C7**	13	Blank	177,4	10,0	118,9	10,2	0,6	0,6
**C7**	13	LLNATIAEV	160,7	4,7	102,6	4,3	0,6	0,6
**P2**	0	Blank	16,4	-	4,0	-	4,6	-
**P2**	0	LLNATIAEV	25,2	-	3,2	-	7,3	-
**P2**	13	Blank	98,5	-	27,4	-	7,3	-
**P2**	13	LLNATIAEV	57,5	-	33,1	-	4,6	-
**P3**	16	Blank	5,7	0,6	8,9	0,0	20,7	3,6
**P3**	16	LLNATIAEV	24,6	0,6	11,0	1,8	178,2	2,9
**P4**	0	Blank	0,9	0,2	5,6	1,2	0,0	0,0
**P4**	0	EPLARLEL	2,0	0,2	6,0	0,6	0,0	0,0
**P4**	13	Blank	18,2	1,8	26,5	1,1	0,0	0,0
**P4**	13	EPLARLEL	27,0	0,2	28,5	0,6	1,4	0,6
**P5**	0	Blank	7,5	-	1,6	-	1,6	-
**P5**	0	LLNATIAEV	25,2	-	0,3	-	3,1	-
**P5**	13	Blank	198,9	-	74,1	-	3,1	-
**P5**	13	LLNATIAEV	150,6	-	92,5	-	0,0	-
**P7**	0	Blank	6,0	0,6	4,3	0,6	0,0	0,0
**P7**	0	LLNATIAEV	8,2	1,0	5,5	0,0	0,0	0,0
**P7**	13	Blank	20,7	2,9	23,8	3,9	0,0	0,0
**P7**	13	LLNATIAEV	32,5	3,0	37,5	2,2	0,0	0,0
**P8**	0	Blank	3,7	-	3,8	-	1,8	-
**P8**	0	EPLARLEL	2,3	-	5,5	-	1,8	-
**P8**	13	Blank	1,8	-	5,5	-	0,0	-
**P8**	13	EPLARLEL	2,3	-	4,7	-	0,0	-

Supplementary Table 3. Overview of multiplex cytokine data (IL-10, Granzyme A and IL-13, quantified as pg/ml) from peptide stimulations.

ID	Day	Stimulant	IL-10	SD	Granzyme A	SD	IL-13	SD
**C1**	0	Blank	0,8	-	101,5	-	#NUM	-
**C1**	0	LLNATIAEV	3,1	-	226,9	-	1,6	-
**C1**	13	Blank	153,4	-	1290,3	-	14,2	-
**C1**	13	LLNATIAEV	167,3	-	1277,3	-	14,2	-
**C3**	0	Blank	13,8	-	931,3	-	2,9	-
**C3**	0	LLNATIAEV	11,6	-	975,6	-	2,2	-
**C7**	0	Blank	24,8	1,6	482,8	0,0	0,3	0,2
**C7**	0	LLNATIAEV	19,1	3,3	555,4	0,0	0,0	0,0
**C7**	13	Blank	43,5	3,0	834,1	21,8	359,1	13,6
**C7**	13	LLNATIAEV	34,8	0,0	699,6	23,1	265,6	17,0
**P2**	0	Blank	11,6	-	807,7	-	2,9	-
**P2**	0	LLNATIAEV	15,9	-	1019,0	-	2,9	-
**P2**	13	Blank	32,5	-	2615,6	-	16,5	-
**P2**	13	LLNATIAEV	32,5	-	2428,9	-	17,0	-
**P3**	16	Blank	0,0	0,0	129,1	10,9	2,4	0,0
**P3**	16	LLNATIAEV	0,0	0,0	261,4	8,3	11,8	0,0
**P4**	0	Blank	11,9	3,4	199,7	9,3	0,0	0,0
**P4**	0	EPLARLEL	8,2	1,8	333,9	15,1	0,0	0,0
**P4**	13	Blank	16,7	3,3	5137,4	234,5	4,6	0,3
**P4**	13	EPLARLEL	16,7	0,0	4312,9	83,2	5,6	1,1
**P5**	0	Blank	5,2	-	582,4	-	1,6	-
**P5**	0	LLNATIAEV	9,5	-	855,0	-	3,5	-
**P5**	13	Blank	42,8	-	2809,4	-	26,5	-
**P5**	13	LLNATIAEV	38,7	-	2397,5	-	20,4	-
**P7**	0	Blank	8,2	1,8	537,6	12,7	0,4	0,0
**P7**	0	LLNATIAEV	13,1	5,1	555,4	0,0	0,7	0,4
**P7**	13	Blank	8,2	1,8	2076,7	719,7	21,8	0,9
**P7**	13	LLNATIAEV	8,2	1,8	1192,9	14,6	25,3	2,1
**P8**	0	Blank	14,3	-	121,4	-	0,9	-
**P8**	0	EPLARLEL	14,3	-	87,7	-	1,5	-
**P8**	13	Blank	23,6	-	633,2	-	1,5	-
**P8**	13	EPLARLEL	14,3	-	267,2	-	2,0	-

Supplementary Table 4. Overview of multiplex cytokine data (Granzyme B, sFAS and IL-2, quantified as pg/ml) from peptide stimulations.

ID	Day	Stimulant	Granzyme B	SD	sFas	SD	IL-2	SD
**C1**	0	Blank	12,1	-	0,0	-	0,1	-
**C1**	0	LLNATIAEV	21,5	-	0,0	-	0,5	-
**C1**	13	Blank	632,2	-	0,0	-	3,2	-
**C1**	13	LLNATIAEV	846,5	-	558,9	-	3,6	-
**C3**	0	Blank	88,0	-	438,4	-	1,0	-
**C3**	0	LLNATIAEV	74,6	-	0,0	-	1,0	-
**C7**	0	Blank	59,8	3,0	3105,3	328,1	0,0	0,0
**C7**	0	LLNATIAEV	54,3	6,2	3072,9	187,7	0,5	0,4
**C7**	13	Blank	1558,7	120,9	2159,0	146,9	37,0	1,6
**C7**	13	LLNATIAEV	1455,8	97,4	2089,6	147,6	28,8	0,4
**P2**	0	Blank	64,1	-	313,3	-	1,4	-
**P2**	0	LLNATIAEV	70,7	-	313,3	-	1,4	-
**P2**	13	Blank	1584,6	-	313,3	-	2,7	-
**P2**	13	LLNATIAEV	1531,5	-	902,7	-	2,3	-
**P3**	16	Blank	110,7	10,7	1590,8	458,6	0,5	0,4
**P3**	16	LLNATIAEV	125,5	2,9	2009,0	841,8	1,7	0,4
**P4**	0	Blank	6,9	0,3	2364,4	339,1	0,0	0,0
**P4**	0	EPLARLEL	11,3	1,9	2603,8	191,5	0,5	0,4
**P4**	13	Blank	3318,2	716,4	2633,6	621,8	1,7	0,4
**P4**	13	EPLARLEL	3401,8	432,5	2804,0	474,5	1,4	0,0
**P5**	0	Blank	21,0	-	31,1	-	1,0	-
**P5**	0	LLNATIAEV	18,7	-	902,7	-	1,0	-
**P5**	13	Blank	1284,8	-	676,0	-	1,8	-
**P5**	13	LLNATIAEV	989,0	-	#NUM	-	1,0	-
**P7**	0	Blank	77,1	2,7	3001,1	753,3	0,2	0,0
**P7**	0	LLNATIAEV	75,3	0,1	3693,7	597,1	0,0	0,0
**P7**	13	Blank	492,6	17,1	1772,1	301,4	0,2	0,0
**P7**	13	LLNATIAEV	354,9	23,5	2397,2	483,7	0,5	0,4
**P8**	0	Blank	13,7	-	1773,7	-	2,4	-
**P8**	0	EPLARLEL	12,6	-	1915,1	-	1,4	-
**P8**	13	Blank	146,0	-	2806,4	-	0,8	-
**P8**	13	EPLARLEL	128,6	-	1631,0	-	1,4	-

Supplementary Table 5. Overview of multiplex cytokine data (IL-4, IL-5 and IL-6, quantified as pg/ml) from peptide stimulations.

**ID**	**Day**	**Stimulant**	**IL-4**	SD	**IL-5**	SD	**IL-6**	SD
**C1**	0	Blank	0,3	-	0,0	-	1,0	-
**C1**	0	LLNATIAEV	0,3	-	0,0	-	4,9	-
**C1**	13	Blank	9,0	-	4,7	-	6,0	-
**C1**	13	LLNATIAEV	7,6	-	5,0	-	7,9	-
**C3**	0	Blank	2,4	-	0,2	-	281,7	-
**C3**	0	LLNATIAEV	2,4	-	0,0	-	321,4	-
**C7**	0	Blank	2,9	1,0	0,0	0,0	126,2	7,0
**C7**	0	LLNATIAEV	2,9	1,0	0,2	0,1	141,8	10,1
**C7**	13	Blank	73,8	0,0	127,7	8,8	0,8	0,5
**C7**	13	LLNATIAEV	57,3	0,9	80,4	7,5	0,3	0,2
**P2**	0	Blank	1,1	-	0,2	-	146,3	-
**P2**	0	LLNATIAEV	1,1	-	0,2	-	206,7	-
**P2**	13	Blank	7,6	-	19,9	-	324,4	-
**P2**	13	LLNATIAEV	9,0	-	21,0	-	22,8	-
**P3**	16	Blank	1,5	1,0	0,1	0,0	6,3	0,2
**P3**	16	LLNATIAEV	12,8	0,0	0,3	0,0	73,8	3,2
**P4**	0	Blank	1,5	1,0	0,3	0,0	4,7	0,4
**P4**	0	EPLARLEL	2,2	0,0	0,4	0,1	36,6	4,0
**P4**	13	Blank	6,3	0,0	2,5	0,0	6,1	0,2
**P4**	13	EPLARLEL	6,3	0,0	2,9	0,3	38,3	1,0
**P5**	0	Blank	2,4	-	0,0	-	12,1	-
**P5**	0	LLNATIAEV	3,7	-	0,0	-	68,2	-
**P5**	13	Blank	11,6	-	10,7	-	19,7	-
**P5**	13	LLNATIAEV	5,0	-	10,7	-	18,3	-
**P7**	0	Blank	2,2	0,0	0,0	0,0	77,5	5,7
**P7**	0	LLNATIAEV	3,6	0,0	0,3	0,0	119,5	11,2
**P7**	13	Blank	3,6	0,0	6,3	1,2	2,1	0,6
**P7**	13	LLNATIAEV	4,3	0,9	6,3	0,4	2,2	0,1
**P8**	0	Blank	2,2	-	0,3	-	161,1	-
**P8**	0	EPLARLEL	0,8	-	0,3	-	168,8	-
**P8**	13	Blank	6,3	-	1,1	-	0,6	-
**P8**	13	EPLARLEL	4,9	-	1,1	-	0,6	-

Supplementary Table 6. Overview of multiplex cytokine data (sFasL, MIP1a and MIP1b, quantified as pg/ml) from peptide stimulations.

ID	Day	Stimulant	sFasL	SD	MIP1a	SD	MIP1b	SD
**C1**	0	Blank	6,6	-	47,2	-	130,8	-
**C1**	0	LLNATIAEV	14,9	-	110,9	-	225,7	-
**C1**	13	Blank	60,4	-	3127,0	-	1858,0	-
**C1**	13	LLNATIAEV	83,0	-	2713,8	-	2079,9	-
**C3**	0	Blank	6,6	-	3912,0	-	2535,0	-
**C3**	0	LLNATIAEV	5,4	-	3982,1	-	2159,0	-
**C7**	0	Blank	2,2	0,0	2902,0	111,3	277,0	11,7
**C7**	0	LLNATIAEV	8,8	11,0	3207,1	187,8	425,9	14,8
**C7**	13	Blank	19,8	0,7	507,0	4,5	829,1	36,3
**C7**	13	LLNATIAEV	9,6	1,4	390,8	13,6	638,7	21,1
**P2**	0	Blank	10,2	-	2745,1	-	2561,3	-
**P2**	0	LLNATIAEV	22,8	-	3755,3	-	3578,3	-
**P2**	13	Blank	48,8	-	1159,7	-	1526,7	-
**P2**	13	LLNATIAEV	57,2	-	1144,9	-	1549,6	-
**P3**	16	Blank	4,4	1,6	167,0	10,4	320,1	17,7
**P3**	16	LLNATIAEV	11,6	1,4	472,1	16,9	807,2	14,4
**P4**	0	Blank	0,0	0,0	352,6	18,3	554,7	5,0
**P4**	0	EPLARLEL	8,1	2,2	925,6	152,8	922,1	143,0
**P4**	13	Blank	17,4	2,7	2196,0	485,4	1026,3	56,1
**P4**	13	EPLARLEL	28,1	4,5	2986,1	180,7	1405,7	20,9
**P5**	0	Blank	11,4	-	1017,2	-	1222,1	-
**P5**	0	LLNATIAEV	17,2	-	3022,1	-	2239,0	-
**P5**	13	Blank	138,9	-	1774,8	-	3339,6	-
**P5**	13	LLNATIAEV	107,2	-	489,1	-	3154,8	-
**P7**	0	Blank	0,0	0,0	3522,0	44,6	881,0	38,9
**P7**	0	LLNATIAEV	1,0	0,0	3731,7	123,2	1297,4	18,7
**P7**	13	Blank	13,6	1,3	546,2	0,6	399,1	24,8
**P7**	13	LLNATIAEV	26,3	3,3	532,2	18,3	1470,5	90,5
**P8**	0	Blank	1,0	-	21,4	-	90,2	-
**P8**	0	EPLARLEL	4,4	-	20,4	-	102,4	-
**P8**	13	Blank	0,0	-	9,4	-	56,6	-
**P8**	13	EPLARLEL	0,0	-	9,4	-	65,4	-

Supplementary Table 7. Overview of multiplex cytokine data (TNFa and Perforin, quantified as pg/ml) from peptide stimulations.

ID	Day	Stimulant	TNFa	SD	Perforin	SD
**C1**	0	Blank	6,7	-	415,2	-
**C1**	0	LLNATIAEV	13,5	-	460,6	-
**C1**	13	Blank	142,0	-	889,8	-
**C1**	13	LLNATIAEV	119,3	-	1085,6	-
**C3**	0	Blank	177,3	-	106,5	-
**C3**	0	LLNATIAEV	193,8	-	86,3	-
**C7**	0	Blank	644,9	1,8	76,1	0,0
**C7**	0	LLNATIAEV	520,8	19,8	85,7	2,6
**C7**	13	Blank	39,0	0,4	448,5	6,2
**C7**	13	LLNATIAEV	32,1	0,1	433,9	6,2
**P2**	0	Blank	404,7	-	319,6	-
**P2**	0	LLNATIAEV	583,3	-	340,1	-
**P2**	13	Blank	526,2	-	1183,5	-
**P2**	13	LLNATIAEV	184,0	-	1303,7	-
**P3**	16	Blank	47,9	2,8	143,3	22,1
**P3**	16	LLNATIAEV	309,0	7,0	190,8	2,4
**P4**	0	Blank	65,0	2,5	261,7	20,1
**P4**	0	EPLARLEL	102,7	11,7	280,6	19,9
**P4**	13	Blank	89,7	4,0	518,7	20,0
**P4**	13	EPLARLEL	138,9	0,1	554,0	13,9
**P5**	0	Blank	94,8	-	174,2	-
**P5**	0	LLNATIAEV	293,1	-	152,0	-
**P5**	13	Blank	76,1	-	3672,8	-
**P5**	13	LLNATIAEV	27,9	-	3139,8	-
**P7**	0	Blank	388,9	31,3	205,6	13,9
**P7**	0	LLNATIAEV	544,8	6,2	258,7	6,7
**P7**	13	Blank	58,8	0,3	300,8	13,1
**P7**	13	LLNATIAEV	51,9	4,7	404,6	18,7
**P8**	0	Blank	23,0	-	352,5	-
**P8**	0	EPLARLEL	24,4	-	361,5	-
**P8**	13	Blank	7,0	-	162,3	-
**P8**	13	EPLARLEL	5,7	-	155,4	-

Supplementary Table 8. Peptide information for REVEAL® MHC peptide binding assay (Module 1).

ID	Peptide Sequence
1	GEPLARLE
2	EPLARLEL
3	PLARLELF
4	LARLELFV
5	ARLELFVV
6	RLELFVVL
7	LELFVVLT
8	ELFVVLTR
9	LFVVLTRL
10	FVVLTRLL
11	VVLTRLLQ
12	GEPLARLEL
13	EPLARLELF
14	PLARLELFV
15	LARLELFVV
16	ARLELFVVL
17	RLELFVVLT
18	LELFVVLTR
19	ELFVVLTRL
20	LFVVLTRLL
21	FVVLTRLLQ

Supplementary Table 9. B*08:01 Binding Data from REVEAL® MHC peptide binding assay (Module 2).

Peptide ID	Peptide Sequence	REVEAL® Score at 0 h
1	GEPLARLE	0,3
2	EPLARLEL	78,0
3	PLARLELF	0,2
4	LARLELFV	0,2
5	ARLELFVV	0,2
6	RLELFVVL	0,1
7	LELFVVLT	0,1
8	ELFVVLTR	0,2
9	LFVVLTRL	0,4
10	FVVLTRLL	32,3
11	VVLTRLLQ	0,1
12	GEPLARLEL	1,8
13	EPLARLELF	82,7
14	PLARLELFV	0,1
15	LARLELFVV	5,1
16	ARLELFVVL	0,3
17	RLELFVVLT	0,0
18	LELFVVLTR	0,1
19	ELFVVLTRL	0,1
20	FVVLTRLLQ	0,2
Positive Control		100,0

Supplementary Table 10. ProVE® Pentamer Library Analysis from REVEAL® MHC peptide binding assay (Module 3).

Peptide I.D.	Peptide Sequence	Concentration (µg/ml)	Volume (µl)	Approximate ul needed per test (0.5 µg/test)
2	EPLARLEL	213,19	360	2,35
13	EPLARLELF	234,56	360	2,13
Positive Control		Passed		

Supplementary Table 11. Summary of the binding and rate data for peptides passing the ProImmune REVEAL® MHC peptide binding assay.

Peptide ID	Peptide Sequence	REVEAL® Score	Off-rate $T_{1/2}$ (h)	Quick Score
2	EPLARLEL	78,00	62,12 ‡	4,85
13	EPLARLELF	82,70	6,93	0,57
Positive Control	~	100,00	> 120,00 ‡ +/- 0,00	12,00

Supplementary Table 12. Additional HLA class I molecules predicted to bind ARLELFVVL and their frequencies in patients and controls.

HLA-Alleles	Frequencies (%)[1]		Frequencies (%)[2]	
	AAD	Controls	AAD	Controls
HLA-A*3207	ND	ND	ND	ND
HLA-B*1402	0.6	2.1	0.4	1.3
HLA-B*2705	5.7	7.2	5.4	5.7
HLA-B*2720	ND	ND	ND	ND
HLA-B*3801	0.5	0.7	0.0	0.5
HLA-B*3901	2.2	1.5	0.5	0.6
HLA-B*4002	NA	NA	0.7	1.6
HLA-B*4801	0.4	0.2	0.1	0.1
HLA-B*7301	ND	0.1	ND	ND
HLA-C*0401	NA	NA	2.9	8.9
HLA-C*0602	NA	NA	1.6	6.7
HLA-C*0702	NA	NA	14.5	15.7

[1] Frequencies taken from Skinningsrud et al (1).
[2] Frequencies taken from Eriksson et al (2)

Accompanying table to Figure 1. Dextramer frequencies expressed as % of total CD8+ T cells

	Day 0	Day 13	Day 0	Day 13
**Subjects**	**A2*LLNATIAEV**	**A2*LLNATIAEV**	**B8*EPLARLEL**	**B8*EPLARLEL**
**C1**	0.009	2.508	-	-
**C2**	-	-	0	0.009
**C3**	0.004	0.006	-	-
**C4**	-	-	0.002	0.007
**C5**	-	-	0.002	0
**C7**	0.003	0.111	-	-
**C8**	0.007	0.071	0	-
**C9**	0.006	0.592	-	-
**P1**	0.013	0.03	-	-
**P2**	0,015	2.455	-	-
**P3**	0.035	16.317	-	-
**P4**	-	-	0.002	0.018
**P5**	0.183	12.142	-	-
**P6**	-	-	0.002	0.002
**P7**	0.053	13.955	-	-
**P8**	-	-	0.007	0.118
**P9**	0.103	-	-	-
**P10**	0.128	2.63	-	-
**P11**	0.006	1.399	-	-
**P12**	-	-	0.008	0.027

Accompanying table to Figure 2. ELISPOT results expressed as IFNγ SFC per 6 x 10^5 PBMC (Blank subtracted)

	**Day 0**	**Day 13**	**Day 0**	**Day 13**
**Subjects**	**LLNATIAEV**	**LLNATIAEV**	**EPLARLEL**	**EPLARLEL**
**C1**	0	468	-	-
**C2**	-	-	6.5	13.5
**C3**	0	-	-	-
**C4**	-	-	1.5	0.5
**C5**	-	-	1	0
**C7**	4	0	-	-
**C8**	0.5	13	-	-
**C9**	0	8	-	-
**P1**	0	5	-	-
**P2**	8	555	-	-
**P3**	2	21	-	-
**P4**	-	-	0	10
**P5**	5.5	455	-	-
**P6**	-	-	0	0
**P7**	19	1952	-	-
**P8**	-	-	0	0
**P9**	6	-	-	-
**P10**	185	239	-	-
**P11**	1	0	3	0
**P12**	-	-	0.5	0

Accompanying table to Figure 3. ELISPOT results expressed as IFNγ SFC per 6 x 10^5 PBMC (Blank subtracted)

	**C12**	**C13**	**C14**	**C15**	**C16**	**P13**	**P14**	**P15**	**P16**	**P17**	**P18**	**P19**	**P20**	**P21**	**P22**
**Pep1**	0	3	8	4	0	0	8	0.5	0	1	4	0	0	4	0
**Pep2**	2	0	0	3	2	0	0.5	0.5	0	3	3	3	2	13	1
**Pep3**	0	0	0	0	0	0	15.5	0	0	7	3	0	0	0	2
**Pep4**	0	0	0	1	8	12.5	14	0.5	0	6	2	5	0	0	1
**Pep5**	0	0	0	5	6	1.5	10	0	15	1	51	8	0	13	1
**Pep6**	0	0	0	1	2	2.5	4.5	0	0	0	0	2	0	0	1
**Pep7**	0	0	0	4	2	7.5	19.5	0	0	3	1	5	0	16	3
**Pep8**	0	0	0	1	5	0	1.5	0	0	0	0	4	1	5	0
**Pep9**	0	0	0	4	3	0	0	0	6	0	1	0	0	2	1
**Pep10**	0	0	0	0	12	1	10	0	0	0	8	0	0	9	3
**P34**	0	0	0	0	0	1.5	10	1	48	0	60	9	16	0	0

Accompanying table to Figure 4. Streptamer frequencies expressed as % of total CD8+ T cells and ELISPOT results expressed as IFNγ SFC per 6 x 10^5 PBMC (Blank subtracted)

	**Streptamer frequencies**		**IFNγ ELISPOT SFC**	
	C7*ARLELFVVL **d0**	C7*ARLELFVVL **d13**	ARLELFVVL	P34
**C17**	0.014	-	0	0
**C18**	0.028	-	0	0
**C19**	0.012	-	0	0
**C20**	0.053	-	0	0
**C21**	0.024	-	0	0
**C22**	0.016	-	0	0
**C23**	0.023	-	0	0
**C24**	0.013	0	0	0
**C25**	0.025	0.011	0	0
**C26**	0.011	0.01	0	0
**C27**	0.018	0.005	1	0
**C28**	0.025	0.052	0	0
**C29**	0.01	-	0	0
**C30**	0.019	-	0	0
**P18**	0.32	-	51	60
**P19**	0.14	1.53	8	9
**P20**	0.075	-	5	6
**P23**	0.024	-	0	0
**P24**	-	-	4	4
**P25**	0.077	-	5	9
**P26**	0.041	-	1	7
**P27**	0.051	-	1	3
**P28**	0.047	-	1	2
**P29**	0.2	1.35	5	26
**P30**	0.067	0.95	7	11
**P31**	-	-	4	1
**P32**	0.012	0.72	0	0
**P33**	0.017	0.5	1	1
**P34**	0.11	0.63	-	-

Supplementary References

1. Skinningsrud B, Lie BA, Lavant E, Carlson JA, Erlich H, Akselsen HE, et al. Multiple loci in the HLA complex are associated with Addison's disease. J Clin Endocrinol Metab. 2011;96(10):E1703-8.
2. Eriksson D, Royrvik EC, Aranda-Guillen M, Berger AH, Landegren N, Artaza H, et al. GWAS for autoimmune Addison's disease identifies multiple risk loci and highlights AIRE in disease susceptibility. Nat Commun. 2021;12(1):959.
